# Oxidative Stress and Antioxidants in Glioblastoma: Mechanisms of Action, Therapeutic Effects and Future Directions

**DOI:** 10.3390/antiox14091121

**Published:** 2025-09-15

**Authors:** Agnieszka Nowacka, Maciej Śniegocki, Ewa Ziółkowska

**Affiliations:** 1 Department of Neurosurgery, Collegium Medicum in Bydgoszcz, Nicolas Copernicus University in Toruń, Ul. Curie Skłodowskiej 9, 85-094 Bydgoszcz, Poland; 2 Department of Pediatrics, School of Medicine, Washington University in St. Louis, St. Louis, MO 63110, USA

**Keywords:** glioblastoma, GB, oxidative stress, reactive oxygen species, ROS, tumor microenvironment, oxidative imbalance, cell death, cancer cells, antioxidants, carotenoids, CAT, coenzyme Q10, curcumin, flavonoids, glutathione, resveratrol, SOD, vitamin A, vitamin E, vitamin C, ROS, oxidative stress

## Abstract

Glioblastoma (GB) is an aggressive and treatment-resistant primary brain tumor with a dismal prognosis. Increasing evidence implicates oxidative stress as a central driver of its pathogenesis, progression, and resistance to therapy. The dynamic interplay between oxidative stress and antioxidant mechanisms is fundamental to understanding GBM biology and shaping novel therapeutic approaches. This review synthesizes current knowledge on the multifaceted role of redox biology in glioblastoma, highlighting the molecular mechanisms through which oxidative stress influences tumor proliferation, survival, immune evasion, and metabolic adaptation. Particular focus is given to the tumor microenvironment, hypoxia-driven reactive oxygen species, redox-regulating enzymes, and the immunosuppressive conditions fostered by oxidative stress. Antioxidants, in this context, demonstrate a dual role: while they can mitigate oxidative damage, their effects on cancer cells and treatment outcomes vary depending on the therapeutic setting. We further examine emerging strategies that target oxidative pathways, including small-molecule inhibitors, redox-modulating agents, and combinatorial approaches with standard treatments, while also addressing the complexities posed by antioxidant interventions. Preclinical and clinical findings are reviewed to underscore both the opportunities and challenges of exploiting redox vulnerabilities in GB. Ultimately, a deeper understanding of oxidative stress dynamics and antioxidant regulation may guide the development of innovative therapies that overcome resistance and improve outcomes for patients facing this devastating malignancy.

## 1. Introduction

Glioblastoma (GB) is the most aggressive and most common malignant primary brain tumor in adults, characterized by its rapid growth, resistance to standard treatments, and unfavorable prognosis [1,2,3,4,5,6,7]. Despite advances in medical science, glioblastoma remains one of the most lethal cancers, with a median survival rate of 12 to 14.6 months after diagnosis [8].

GB often manifests with symptoms like headaches, seizures, confusion, and focal neurological deficits, which tend to worsen over time [4,9]. Magnetic Resonance Imaging is a key diagnostic tool, typically showing heterogeneously enhancing tumors located in the subcortical white matter [10,11]. A definitive diagnosis is made through histological examination of surgically obtained tissue, revealing an infiltrating astrocytic tumor, potentially with areas of necrosis and/or endothelial proliferation [12,13].

Standard treatment involves surgical resection followed by radiotherapy and chemotherapy [1,3,4,6,8,14]. The initial therapeutic approach for glioblastoma involves surgical resection, aiming to remove as much of the tumor mass as feasible; however, the infiltrative nature of glioblastoma often precludes complete removal. Recent advancements in molecular pathology have identified key pathways and genetic mutations, such as EGFR and PTEN alterations, which are potential targets for therapy [13]. Emerging therapeutic approaches can be broadly categorized into targeted therapies and immunotherapies. Targeted therapies focus on specific molecular pathways dysregulated in glioblastoma, including inhibitors targeting EGFR and VEGF pathways, as well as PI3K pathway modulators [3,4,8,12,13,15]. Immunotherapeutic strategies aim to harness the immune system against tumor cells, with approaches such as immune checkpoint inhibitors and CAR-T cell therapies showing promise [4,8,12,13,15]. These treatments are often used in combination to minimize side effects and enhance antitumor immune responses. Moreover, a growing number of research has focused on the role of oxidative stress and the antioxidant defense system in the pathophysiology and treatment resistance of GB. It should be noted that glioblastomas, by current WHO classification, are IDH-wildtype tumors; the presence of an IDH mutation instead defines astrocytoma, IDH-mutant, or oligodendroglioma, IDH-mutant and 1p/19q codeleted, entities with distinct biology and prognosis [16]. While IDH mutations are not present in glioblastoma, they remain relevant in the context of oxidative stress in IDH-mutant gliomas, as the mutant IDH enzyme produces 2-hydroxyglutarate, which affects cellular redox homeostasis [17].

## 2. Molecular Basis of Oxidative Stress

**Figure 1:**
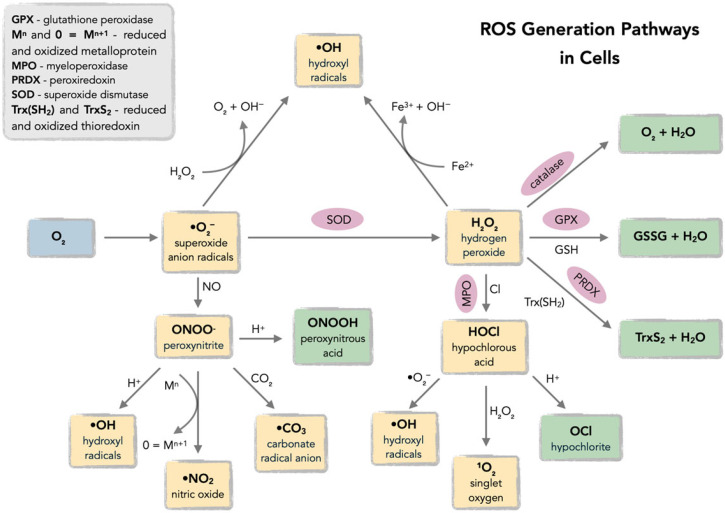
Pathways of reactive oxygen species (ROS) generation in cells, including mitochondrial electron transport chain, NADPH oxidases (NOX), and exogenous sources (ionizing radiation, xenobiotics). Acronyms: ETC—electron transport chain; NOX—NADPH oxidase.

**Figure 2:**
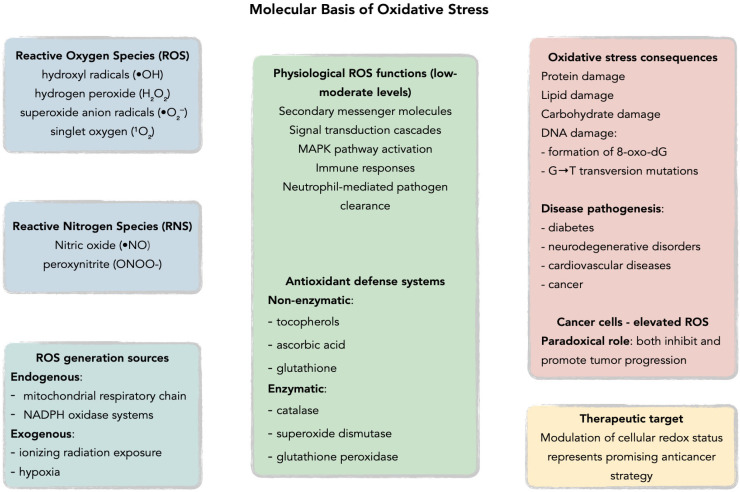
Molecular basis of oxidative stress: imbalance between ROS and antioxidant defenses (SOD, CAT, GPx, GSH, vitamins). Acronyms: ROS—reactive oxygen species; SOD—superoxide dismutase; CAT—catalase; GPx—glutathione peroxidase; GSH—glutathione.

Reactive oxygen species (ROS), defined as oxygen-based free radicals and non-radical derivatives of O_2_ including hydrogen peroxide (H_2_O_2_), superoxide anion radicals (•O_2_^−^), hydroxyl radicals (•OH), and singlet oxygen (^1^O_2_), play a critical dual role in cellular physiology and pathology (Figure 1 and Figure 2) [18,19,20,21,22,23,24,25].

Under physiological conditions, ROS are maintained at appropriate levels by endogenous antioxidant defenses comprising non-enzymatic antioxidants such as tocopherols, ascorbic acid, and glutathione, alongside antioxidant enzymes including catalase, superoxide dismutase, and glutathione peroxidase, with brain tissue demonstrating particularly robust antioxidant systems due to its high metabolic rate and susceptibility to oxidative damage [20,21,24,25,26,27,28,29,30,31,32,33,34]. ROS generation occurs through both endogenous pathways, primarily via the mitochondrial respiratory chain and NADPH oxidase systems, and exogenous processes including ionizing radiation exposure [20,23,25,30,32,35,36,37,38]. At low to moderate concentrations, ROS function as secondary messenger molecules in signal transduction cascades, activating the mitogen-activated protein kinase (MAPK) family pathways and promoting physiological processes such as immune responses through neutrophil-mediated pathogen clearance [30,33,34,39,40,41,42,43]. Specifically, H_2_O_2_ can activate p38 MAPK and JNK pathways in macrophages by oxidatively modifying upstream kinases and inhibiting MAPK phosphatases, leading to nuclear factor-κB (NF-κB) activation and subsequent pro-inflammatory cytokine production—a crucial mechanism in innate immune responses [44,45]. However, when oxidative balance is disrupted, excessive ROS production leads to oxidative stress, resulting in cellular damage through DNA mutations, particularly the formation of 8-oxo-7,8-dihydro-2′-deoxyguanosine (8-oxo-dG) which promotes G→T transversion mutations [23,33,46,47,48,49,50,51]. This oxidative dysregulation has been implicated in the pathogenesis of multiple human diseases including diabetes, cancer, neurodegenerative disorders, and cardiovascular diseases [32,46,47,52,53,54,55,56]. Cancer cells exhibit particularly elevated ROS levels due to aberrant signaling pathways that paradoxically both inhibit and promote tumor progression, suggesting that therapeutic modulation of cellular redox status represents a promising avenue for anticancer strategies [32,46,47,57]. Glioblastoma cells demonstrate a distinct ROS profile characterized by elevated NADPH oxidase 4 (Nox4) expression, which drives both tumor invasion and angiogenesis, setting them apart from other solid tumors where different ROS-generating systems predominate [58,59].

## 3. Oxidative Stress in Glioblastoma

Oxidative stress significantly contributes to the pathogenesis and progression of GBM by influencing tumor cell proliferation, survival, and therapeutic resistance [57,59,60,61,62,63,64]. Reactive oxygen species, as cellular oxidative metabolites, not only promote the development of gliomas but also affect immune cells in the tumor microenvironment [59,60,61,63]. The balance of ROS is crucial, as excessively high or low levels can be detrimental to the survival of glioma cells [59,60,61,63,65]. GBM cells often adapt to high ROS levels, paradoxically enhancing their resistance to therapies [66]. Therapies like chemotherapy and radiation can induce oxidative stress, initially killing tumor cells but potentially leading to the selection of more resistant GSCs, which can result in tumor recurrence and progression [65]. Targeting the redox balance and oxidative stress pathways could be a potential strategy for improving treatment outcomes in glioblastoma [65].

### 3.1. Clinical and Experimental Evidence of Oxidative Stress in GBM

The study by Faraji-Rad et al. provides compelling evidence of oxidative stress in patients with glioblastoma [67]. The study involved 50 patients diagnosed with glioblastoma and 49 healthy subjects [67]. The pro-oxidant-antioxidant balance (PAB) assay, a colorimetric method that simultaneously measures both pro-oxidant and antioxidant activities in serum, revealed a significant increase in pro-oxidant values in glioblastoma patients, with a mean value of 158.10 ± 85.71 HK units, compared to 74.54 ± 33.54 HK units in the control group [67]. HK units represent the standard measurement units for the PAB assay, where higher values indicate greater pro-oxidant activity relative to antioxidant capacity. The statistical analysis confirmed that the difference in pro-oxidant levels between the two groups is statistically significant, with a *p*-value of 0.001 [67]. The study concludes that there is a high level of pro-oxidants present in the sera of patients with glioblastoma multiforme, suggesting the existence of oxidative stress in this patient population [67]. These results support the hypothesis that an imbalance between pro-oxidant factors and antioxidant defenses is prevalent in patients with high-grade gliomas, potentially contributing to the development and progression of these tumors [67]. The pro-oxidant-antioxidant balance assay may be a useful tool for assessing oxidative stress in patients with glioblastoma, which may have implications for understanding the disease’s pathology and developing therapeutic strategies [67].

### 3.2. Hypoxic Microenvironment and ROS

Zhang et al. highlight the critical role of reactive oxygen species in glioblastoma progression, especially within a hypoxic microenvironment [68]. Elevated ROS levels are associated with increased cell proliferation, migration, and invasion of glioblastoma cells [68]. Hypoxia, a common characteristic of the tumor microenvironment, promotes ROS production, exacerbating malignant tumor behavior [68]. Interestingly, the effects of hypoxia on glioblastoma cells can be inhibited by ROS scavengers like N-acetyl-l-cysteine and diphenyleneiodonium chloride [68]. Mechanistically, hypoxia-induced ROS activate the hypoxia-inducible factor-1α (HIF-1α) signaling pathway, enhancing cell migration and invasion through epithelial–mesenchymal transition [68]. The research also indicates that under hypoxic conditions, ROS upregulate the expression of SERPINE1, mediated by HIF-1α binding to the SERPINE1 promoter region, further facilitating glioblastoma cell migration and invasion [68]. Overall, these findings suggest that targeting ROS could be an effective strategy for glioblastoma treatment by disrupting the HIF-1α-SERPINE1 signaling pathway that promotes tumor progression in hypoxic environments [68].

In glioblastoma, tumor hypoxia leads to increased production of hydrogen peroxide (H_2_O_2_), which is a reactive oxygen species [69]. This accumulation can promote malignant progression and resistance to therapies, highlighting the importance of managing oxidative stress in these tumors [69]. Glutathione peroxidase 1 (GPx1) is an antioxidant enzyme that plays a crucial role in detoxifying H_2_O_2_ [69]. GPx1 is essential for maintaining the balance of H_2_O_2_ in hypoxic glioblastoma [69]. When GPx1 is depleted, there is an overload of H_2_O_2_, leading to increased apoptosis in glioblastoma cells [69]. The depletion of GPx1 not only results in H_2_O_2_ overload but also inhibits the growth of glioblastoma xenografts, indicating that GPx1 is vital for tumor survival under hypoxic conditions [69]. Hypoxia increases the expression of exosomal GPx1, which assists glioblastoma and endothelial cells in countering H_2_O_2_ or radiation-induced apoptosis in vitro and in vivo [69]. GPx1 expression was positively correlated with tumor grade, expression of HIF-1α (hypoxia-inducible factor-1α), HIF-1α target genes, and tetraspanin exosomal marker genes (CD9, CD63, CD81)-conversely, it was inversely correlated with the overall survival outcome in human glioblastoma specimens [69].

### 3.3. Hormesis and the Paradoxical Role of ROS

The concept of hormesis offers an important framework for understanding the complex role of reactive oxygen species in glioblastoma. Hormetic theory suggests that low levels of ROS or other stressors activate protective defense pathways, while excessive ROS induces cell death [70,71]. This creates a biphasic response where mild oxidative stress can promote cellular survival and adaptation, but higher levels become cytotoxic [72]. In glioblastoma, this relationship is particularly nuanced. GB cells exist under chronic oxidative stress shaped by evolutionary pressures, enabling them to exploit moderate ROS for proliferation, invasion, and resistance to therapy [73]. This aligns with evidence that reduced glutathione (GSH) levels correlate with increased tumor aggressiveness, as diminished antioxidant defenses may encourage tumor growth and recurrence. The hormetic model highlights a critical therapeutic challenge: insufficient ROS can fuel tumor progression through adaptive mechanisms, while excessive ROS can trigger apoptosis [74]. This has direct implications for antioxidant supplementation and pro-oxidant therapies, where the timing, dosage, and combinations must be carefully optimized to push GB cells past their adaptive threshold without harming normal brain tissue. Within this framework, the paradoxical pro-oxidant actions of compounds like lycopene and CoQ10 in cancer cells, alongside their antioxidant protection in healthy cells, become mechanistically consistent.

### 3.4. Gene Expression Changes Related to Oxidative Stress and Immunosuppression

Liang et al. demonstrated through their research that glioblastoma patients could be categorized based on the expression profiles of oxidative-stress-responsive genes [75]. It was observed that cluster 2, characterized by elevated levels of oxidative stress signaling, correlated with a less favorable prognosis for these patients, underscoring the importance of oxidative stress in the advancement of glioblastoma [75]. Functional and immune analyses indicated an elevated presence of M2-like pro-tumoral macrophages and neutrophils within cluster 2 [75]. Conversely, the infiltration of Natural Killer cells was notably diminished [75]. This alteration in immune cell composition suggests that oxidative stress may foster an immunosuppressive milieu in glioblastoma [75]. Immunofluorescence analyses corroborated the presence of M2-like pro-tumoral macrophages within cluster 2 [75]. Moreover, comprehensive single-cell analysis further substantiated the malignant attributes of the neoplastic cells in this cluster and emphasized their interplay with M2-like macrophages [75]. The study also examined the function of Superoxide Dismutase 3 (SOD3) in modulating macrophage activity [75]. The results indicated that the suppression of SOD3 expression could diminish the differentiation of macrophages into the M2-like pro-tumoral phenotype, both in vitro and in vivo [75]. These results imply that SOD3 may represent a viable target for therapeutic interventions designed to modulate the immune response in glioblastoma [75]. In conclusion, oxidative stress plays a pivotal role in glioblastoma, impacting both the advancement of the tumor and the immunological environment. These results highlight the therapeutic potential of targeting oxidative stress pathways, with a particular focus on SOD3, to enhance the efficacy of immunotherapy for individuals afflicted with glioblastoma.

### 3.5. SP/NK1R Pathway as a Source of Oxidative Stress

Substance P (SP) is an 11-amino acid neuropeptide that functions as both a neurotransmitter and neuromodulator in the central nervous system [76,77]. SP exerts its biological effects primarily through binding to the neurokinin-1 receptor (NK1R), a G-protein coupled receptor that is widely distributed throughout the body [76]. The SP/NK1R signaling pathway is involved in various physiological processes including pain perception, inflammation, and stress responses [76]. In the context of cancer, SP binding to NK1R activates downstream signaling cascades including PI3K/Akt/NF-κB pathways that promote tumor cell proliferation, migration, invasion, and angiogenesis while inhibiting apoptosis [78,79]. The SP/NK1R system has been identified as a promising therapeutic target, with NK1R antagonists showing potential as anticancer treatments across various tumor types [78].

Rezaei et al. investigated the role of substance P (SP) and neurokinin 1 receptor (NK1R) in glioblastoma cells [80]. Activating the SP/NK1R axis increases oxidative stress, potentially contributing to tumor progression [80,81]. The study found that SP increased levels of malondialdehyde and reactive oxygen species in U87 glioblastoma cells, while aprepitant, an NK1R antagonist, reduced cell survival and had antioxidant properties [80]. The concurrent administration of aprepitant and substance P led to a notable decrease in oxidative stress markers, suggesting that aprepitant is capable of modulating the balance between oxidants and antioxidants [80]. These results imply that the SP/NK1R signaling pathway is significantly involved in the augmentation of oxidative stress within glioblastoma cells, potentially contributing to the advancement of the tumor [80]. Conversely, aprepitant seems to have anticancer properties by diminishing oxidative stress and bolstering antioxidant mechanisms in these cells [80]. In summary, targeting the SP/NK1R axis through the use of aprepitant may represent a viable approach for creating novel glioblastoma treatments, as it has the potential to regulate oxidative stress and thereby enhance patient outcomes.

These findings align with the understanding that the SP/NK1R signaling pathway promotes oxidative stress in glioblastoma cells [81]. Substance P increased ROS levels in U87 glioblastoma cells, an effect that aprepitant significantly reduced [81]. Furthermore, SP could also affect the thioredoxin system, a central antioxidant enzyme defense system [81]. Substance P reduced both expression and enzymatic activity of the thioredoxin system’s proteins, Trx and thioredoxin reductase, and these effects were significantly reduced by aprepitant [81]. This links NK1R signaling directly to suppression of the thioredoxin system, thereby increasing oxidative stress and sensitizing glioblastoma cells to redox modulation. These findings suggests that SP activation of NK1R represents a link between oxidative stress and GBM [81].

Mehrabani et al. aimed to examine how substance P and aprepitant, an NK1R antagonist, influence redox processes within glioblastoma cells [82]. The findings revealed that SP elevates intracellular ROS levels in U87 GBM cells, an effect significantly mitigated by aprepitant [82]. The study also investigated how SP/NK1R signaling affects the glutaredoxin system, a major cellular redox buffer. SP reduced both glutaredoxin expression and enzymatic activity, changes that aprepitant lessened [82]. In conclusion, the results suggest SP/NK1R signaling may contribute to GBM development through oxidative stress, highlighting aprepitant as a potential redox-modulating strategy for GBM patients [82].

Korfi et al. assessed the SP/NK1R system’s influence on catalase and superoxide dismutase expression and activity in the U87 glioblastoma cell line [83]. The study found that aprepitant reduced U87 cell viability in a concentration-dependent manner and significantly reduced ROS production and increased catalase and SOD activity [83]. The authors concluded that aprepitant inhibits SP’s oxidizing effects by inducing the antioxidant effects of catalase and SOD in the U87 cell line, suggesting it as a potential candidate for controlling glioblastoma in animal models and clinical trials [83].

### 3.6. Oxidative Stress in Metabolism, Invasion, and Motility

Treatment with oxidants like amyloid beta peptide, glucose oxidase, and hydrogen peroxide increases the expression and activity of Apurinic/apyrimidinic endonuclease (APE1), a crucial enzyme in DNA base excision repair, as well as glycolytic enzymes like Pyruvate kinase M2 and Ectonucleotide pyrophosphatase/phosphodiesterase 2 (ENPP2) [84]. The upregulation of these glycolytic enzymes under oxidative stress conditions contributes to cancer cell survival and migration through several interconnected mechanisms. PKM2, beyond its role as a rate-limiting glycolytic enzyme, functions as a protein kinase that promotes tumorigenesis by facilitating metabolic reprogramming from normal oxidative phosphorylation to aerobic glycolysis (the Warburg effect), which provides cancer cells with rapid ATP generation and biosynthetic precursors necessary for proliferation and survival [85,86]. Secreted PKM2 induces cell migration via activating the PI3K/Akt and Wnt/β-catenin signaling pathways, directly linking glycolytic enzyme upregulation to enhanced migratory capacity [87]. Cancer cells adapt to oxidative stress by metabolic reprogramming, resulting in cancer residuality, progression, and relapse, which is highly dependent on NADPH and GSH syntheses for ROS scavenging [88]. Similarly, ENPP2 (autotaxin) promotes cancer cell survival and migration by producing lysophosphatidic acid (LPA), a bioactive lipid mediator that promotes tumor cell migration and metastasis via LPAR1 [89]. Autotaxin is a potent stimulator of cell migration, invasion, metastasis, and angiogenesis [90]. The extracellular secretion of APE1 and ENPP2 induced by oxidative stress indicates a potential role for these proteins outside the cell, where they may function as paracrine signaling molecules that enhance the invasive tumor microenvironment [84]. The crosstalk between APE1, PKM2, and ENPP2, mediated by oxidative stress, may play a critical role in the invasive potential of glioblastoma cells by simultaneously promoting DNA repair capacity, metabolic flexibility, and pro-migratory signaling cascades [84].

Orlicka-Płocka et al. found that purine derivatives like Kinetin riboside (KR) and its derivatives (8-azaKR, 7-deazaKR) induce oxidative stress in glioblastoma multiforme cells [66]. This oxidative impairment affects the redox status of cancer cells, which is vital for their growth and survival [66]. KR and 7-deazaKR increase reactive oxygen species levels in T98G cells, contributing to cellular damage and inducing apoptosis [66]. GBM cells typically have increased basal levels of ROS, helping them survive and develop resistance to treatments [66]. By using KR and its derivatives, researchers aimed to surpass the antioxidant defenses of these cancer cells, making them more susceptible to oxidative stress [66]. The compounds not only generated ROS but also caused lipid peroxidation, leading to apoptosis in the T98G cells [66]. The findings suggest that KR and 7-deazaKR could serve as promising alternatives in the oxidative therapy of GBM by manipulating the redox status of cancer cells [66].

Saurty-Seerunghen et al. found that glioblastoma cells with high motile potential exhibit increased levels of reactive oxygen species [91]. These cells also had a higher mitochondrial mass, linked to increased energy production, which is seemingly necessary for their movement [91]. The enzyme 3-Mercaptopyruvate sulfurtransferase (MPST) was identified as crucial for managing oxidative stress in GBM cells, protecting protein cysteine residues from hyperoxidation [91]. Knocking down MPST led to reduced cell motility and increased oxidative stress, indicating its protective role [91]. The research also highlighted that motile GBM cells are enriched in metabolic pathways that counteract oxidative stress, such as the pentose phosphate pathway and glutathione metabolism, suggesting these cells have adapted to manage the oxidative stress associated with their high energy demands [91]. Reducing MPST expression not only decreased cell motility but also led to a significant reduction in tumor burden in animal models, indicating that the ability to manage oxidative stress is linked to the malignancy of GBM cells and their overall survival [91].

### 3.7. The Role of Oxidative Stress in Treatment Resistance

In drug-resistant glioblastoma cells, increased oxidative stress is observed, contributing to the challenges in cancer therapies [92]. This heightened oxidative stress can lead to cellular dysfunctions and promote resistance mechanisms [92]. Tiek et al. indicate an enhancement in mitochondrial function within drug-resistant GBM cells [93]. Altered mitochondrial function can exacerbate oxidative stress levels, further complicating treatment responses [92]. Increased oxidative stress correlates also with elevated gamma-glutamylcyclotransferase (GGCT) [92]. GGCT is involved in the production of glutathione, a key antioxidant that helps mitigate oxidative stress [92]. The byproduct of GGCT, pyroglutamic acid, binds to aggregating proteins, indicating a potential mechanism through which oxidative stress and protein aggregation are linked [92]. Observations in recurrent GBM patient samples support these findings, with increased levels of protein aggregation, GGCT expression, and pyroglutamic acid staining noted, suggesting clinical relevance [93].

Dico et al. investigated the mechanisms behind Temozolomide (TMZ) resistance in glioblastoma cells, focusing on the role of mitochondrial-derived oxidative stress and Chaperone-Mediated Autophagy (CMA) activation [93]. The research highlights that sensitive GBM cells show increased cytoplasmic reactive oxygen species levels and CMA activation upon TMZ treatment, leading to cell toxicity [93]. In TMZ-sensitive glioblastoma cells, treatment with temozolomide resulted in a significant increase in cytoplasmic ROS levels, which correlated with the induction of CMA [93]. Conversely, in TMZ-resistant GBM cells, temozolomide treatment did not lead to an increase in cytoplasmic ROS levels or CMA activation, preventing the cytotoxic effects of TMZ [93]. The study also found that by enhancing oxidative stress with hydrogen peroxide (H_2_O_2_) treatments, CMA activation could be recovered in resistant cells, which in turn restored cell cytotoxicity, especially when combined with temozolomide treatment [93]. These findings provide novel insights into the relationship between mitochondrial ROS release, CMA activation, and TMZ responsiveness in GBM [93]. They emphasize the importance of oxidative stress in overcoming resistance to therapy, suggesting that targeting these pathways could improve treatment outcomes for patients with GBM [93].

Additional evidence from recent literature further substantiates the critical role of oxidative stress in glioblastoma treatment resistance. Research has demonstrated that glioblastoma stem cells (GSCs) exhibit distinct redox characteristics that contribute significantly to therapeutic resistance. These stem-like cells maintain lower baseline ROS levels compared to non-stem tumor cells, which enhances their survival under therapeutic stress conditions [94]. The dual nature of oxidative stress in glioblastoma presents a therapeutic paradox: while moderate ROS levels promote tumor growth and resistance, excessive oxidative stress can induce cell death [65].

Mechanistically, TMZ resistance is intimately connected to lipid peroxidation processes, where cytotoxicity is mediated by aldehydes resulting from lipid peroxidation, and aldehyde dehydrogenase 1A3 (ALDH1A3) reduces the number of these toxic aldehydes, thereby conferring resistance [95]. Furthermore, TMZ-induced damage triggers multiple stress responses including DNA damage, oxidative stress, endoplasmic reticulum stress, and metabolic disruption, which collectively activate autophagy through various signaling pathways such as the ATM/AMPK/ULK1 axis and the PI3K/AKT/mTOR pathway [96].

The therapeutic implications of these findings are profound, as evidenced by recent studies showing that cyclooxygenase-2 (COX-2) inhibition can counteract TMZ resistance by inducing oxidative stress and disrupting redox homeostasis [97]. Similarly, innovative approaches using compounds like piperlongumine have demonstrated the ability to overcome TMZ chemoradiotherapy resistance by boosting oxidative stress-inflammation-CD8+ T cell immunity [98]. These therapeutic strategies highlight the potential of targeting redox pathways to enhance treatment efficacy.

Recent systematic analyses have revealed that glioblastoma’s resistance mechanisms are multifaceted, involving rapid growth, molecular heterogeneity, invasive potential, and regenerative capabilities of drug-resistant cancer stem cells [99]. The modulation of oxidative stress through various therapeutic strategies, including degradation of oxidized proteins, glutathione depletion, and inhibition of key signaling pathways like EGFR/AKT, represents promising approaches for combating treatment resistance [94].

### 3.8. Potential Molecular Targets and Redox Pathways

Thioredoxin domain-containing protein 12 (TXNDC12) belongs to the thioredoxin superfamily and possesses a characteristic thioredoxin fold with a consensus active-site sequence (CxxC), playing crucial roles in redox regulation and defense against oxidative stress [100]. As a small, disulfide-containing protein, TXNDC12 functions mechanistically to inhibit lipid peroxidation and ferroptosis, acting in a GPX4-independent manner to maintain cellular redox homeostasis [101]. Members of the thioredoxin superfamily are involved in the refolding of disulfide-containing proteins and regulation of transcription factors, making them essential components of the cellular antioxidant defense system [102]. The sulfoxide-domain containing protein 12 (TXNDC12) is indeed crucial for maintaining the balance between oxidation and reduction, which is vital for the progression of GBM [103]. Reducing TXNDC12 expression led to a significant decrease in cell proliferation in U251 and A172 GBM cell lines [103]. This suggests that TXNDC12 plays a key role in regulating oxidative stress levels in these cells [103]. The knockdown of TXNDC12 resulted in an imbalance in the oxidation-reduction dynamics within GBM cells, indicative of increased oxidative stress, which can hinder cell proliferation and potentially lead to cell death [103]. In addition to in vitro studies, in vivo experiments using stable A172 cells demonstrated a reduction in tumor growth when TXNDC12 expression was modulated, further supporting the idea that inducing oxidative stress through TXNDC12 manipulation can be a viable strategy for treating glioblastoma [103].

Long non-coding RNAs (lncRNAs) have emerged as critical regulators of cellular processes, including oxidative stress response pathways [104]. These non-protein-coding transcripts function as molecular scaffolds and regulatory elements that can modulate the expression of oxidative stress-related genes and influence cellular redox homeostasis through various epigenetic and post-transcriptional mechanisms [104]. Long non-coding RNAs (lncRNAs) have been foung to play a significant role in regulating oxidative stress through various pathways [105]. The study by Shi et al. identified 3073 oxidative stress-related (ORLs) lncRNAs by analyzing RNA sequencing data from glioblastoma and low-grade glioma patients [105]. A prognostic signature consisting of six specific ORLs was developed, shown to be predictive of patient outcomes in glioma, indicating that they could serve as important biomarkers for assessing prognosis [105]. Patients in the high-risk subgroup (based on the ORLs signature) exhibited significant immune cell infiltration, particularly of macrophages and cancer-associated fibroblasts, associated with poorer prognosis, suggesting that oxidative stress may influence the tumor immune microenvironment [105].

Src kinases are non-receptor tyrosine kinases that play fundamental roles in cellular signaling and have been implicated in oxidative stress regulation through multiple mechanisms [106]. These kinases can modulate ROS production and cellular antioxidant responses, influencing cancer cell survival and proliferation. The aberrant activation of Src kinases contributes to tumor progression by altering cellular redox balance and promoting oxidative stress-mediated signaling cascades [106]. Kostić et al. investigated the pro-oxidative effects of two specific Src tyrosine kinase inhibitors, Si306 and its prodrug pro-Si306, on patient-derived glioblastoma cells [107]. Both Si306 and pro-Si306 significantly increased the levels of reactive oxygen species in human glioblastoma cells, specifically in U87 and patient-derived GBM-6 cells [107]. The treatment with both inhibitors also resulted in elevated expression levels of antioxidant enzymes, superoxide dismutase 1 and superoxide dismutase 2 [107]. This suggests that the cells were responding to the increased oxidative stress by attempting to counteract the effects of ROS. The study found that the increase in ROS was accompanied by a disruption in mitochondrial membrane potential [107]. This disruption is often a precursor to cell death, indicating that the inhibitors not only induce oxidative stress but also compromise mitochondrial function. The elevated ROS levels led to double-strand DNA breaks in GBM-6 cells, which is a significant form of DNA damage [107]. This damage is associated with cell death and senescence, ultimately resulting in necrosis [107]. Overall, the pro-oxidative properties of these Src kinase inhibitors present a potential avenue for enhancing the effectiveness of glioblastoma treatment in clinical settings [107].

Nucleoside diphosphate-linked moiety X-type motif 1 (NUDT1), also known as MTH1, sanitizes oxidized purine nucleotides such as 8-oxo-dGTP to prevent their incorporation into DNA, and as highlighted by Tong et al., plays a significant role in regulating oxidative stress and maintaining mitochondrial function in glioblastoma cells, which could have significant implications for future therapies [108]. When NUDT1 was knocked down in GBM cells, there was a notable increase in mitochondrial ROS production [108]. This suggests that NUDT1 plays a protective role against oxidative stress in these cells [108]. Furthermore, the reduction in NUDT1 expression in glioblastoma cells led to a marked increase in overall reactive oxygen species levels [108]. This underscores the importance of NUDT1 in maintaining cellular redox homeostasis [108]. The study also revealed that NUDT1 knockdown resulted in increased malondialdehyde levels, indicative of lipid peroxidation [108]. This observation confirms that NUDT1 is essential for preventing oxidative stress-induced damage to cell membranes in GBM cells [108]. The increase in oxidative stress due to NUDT1 knockdown resulted in impaired mitochondrial function, which is essential for cell survival and proliferation [108]. This impairment can lead to cell death, highlighting the importance of NUDT1 in maintaining mitochondrial health and preventing oxidative damage [108].

Monoamine oxidase B (MAO-B) is a FAD-dependent mitochondrial enzyme located in the outer mitochondrial membrane that catalyzes the oxidative deamination of various biogenic amines, including dopamine [109,110]. During this enzymatic process, MAO-B generates hydrogen peroxide (H_2_O_2_) as a natural byproduct, making it a significant endogenous source of reactive oxygen species within cells [85,109]. The enzyme’s role in ROS generation has positioned it as a key player in oxidative stress-related pathophysiology, particularly in neurodegenerative diseases and cancer [85,110]. Marconi et al. investigated the effects of two novel Monoamine Oxidase B inhibitors, Cmp3 and Cmp5, on oxidative stress in glioblastoma cells [111]. The findings indicate that both Cmp3 and Cmp5 significantly increase the production of ROS in C6 glioma cells, leading to oxidative stress, which can cause cell damage and death [111]. The induction of oxidative stress by these MAO-B inhibitors resulted in cell cycle arrest, specifically either a G1 or G2/M phase arrest in the glioma cells, preventing their proliferation [111]. The treatment with Cmp3 and Cmp5 also led to a depolarization of the Mitochondrial Membrane Potential, often associated with increased oxidative stress and apoptosis, further contributing to the death of glioma cells [111]. Furthermore, inhibitors reduced the expression levels of inducible nitric oxide synthase 2 (iNOS2), potentially helping manage oxidative stress levels [111]. Notably, Cmp5 was effective in reducing glioma cell migration by downregulating Matrix Metalloproteinases 2 and 9, which is likely linked to the increased oxidative stress and subsequent cell cycle arrest [111]. Overall, the results suggest that the novel MAO-B inhibitors Cmp3 and Cmp5 significantly upregulate oxidative stress in glioblastoma cells, leading to cell cycle arrest and reduced cell migration, which may enhance their therapeutic potential against this aggressive cancer [111].

### 3.9. Therapeutic Strategies Exploiting Oxidative Stress

In their study, Cesca et al. assessed basal levels of reactive oxygen species and the expression of antioxidant genes across various GBM cell lines, showing significant variability likely due to genetic differences [112]. They found that both doxorubicin and photodynamic therapy could effectively induce oxidative stress in glioblastoma cells, leading to increased cytotoxicity, potentially overcoming resistance to conventional therapies [112]. The study concludes that pro-oxidant therapies, particularly the combination of doxorubicin and PDT, could selectively target GBM cells by exploiting their elevated oxidative stress levels, presenting a promising strategy for improving therapeutic outcomes in patients with glioblastoma [112].

Loenhout et al. investigated the effects of auranofin (AF) and cold atmospheric plasma (CAP) on glioblastoma cells, focusing on the generation of reactive oxygen species as a therapeutic strategy [113]. The combination treatment significantly increased the levels of exogenous ROS in GBM cells, which is crucial for inducing cell death mechanisms [113]. The combination of AF and cold atmospheric plasma-treated PBS resulted in the highest accumulation of intracellular ROS compared to either treatment alone [113]. This increase in ROS was linked to a decrease in the activity of thioredoxin reductase and glutathione levels, which are important components of the cell’s antioxidant defense system [113]. The study found that the combination treatment led to distinct cell death mechanisms [113]. Specifically, the increase in ROS was associated with apoptosis, indicated by elevated caspase-3/7 activity and a higher proportion of annexin V positive cells, and ferroptosis, with evidence of lipid peroxidation and the ability to inhibit cell death through an iron chelator [113]. Furthermore, the increase in ROS levels and the subsequent cell death mechanisms resulted in the release of danger signals such as ecto-calreticulin, ATP, and HMGB1, which are important for enhancing immunogenic responses, indicating that the treatment may also stimulate the immune system [113]. In vivo experiments demonstrated that the sequential combination treatment of AF and cold atmospheric plasma reduced tumor growth and prolonged survival in GBM-bearing mice, further supporting the therapeutic potential of targeting ROS in glioblastoma treatment [113].

Burccarelli et al. identified elesclomol as a potent inducer of oxidative stress, particularly in glioblastoma stem-like cells (GSCs) and GSC-derived endothelial cells (GdECs) [114]. Elesclomol significantly increases mitochondrial reactive oxygen species levels, which can damage cells and contribute to cell death [114]. It induces a unique form of cell death that is copper-dependent and non-apoptotic [114]. Moreover, it promotes the production of mitochondrial ROS, leading to alterations in mitochondrial membrane potential and a decrease in glutathione levels, disrupting the balance of oxidative stress within the cells [114]. The increase in oxidative stress due to elesclomol treatment results in a dose-dependent decrease in cell viability for both GSCs and GdECs [114]. When elesclomol was combined with temozolomide, it enhanced the cytotoxic effects compared to either treatment alone [114]. This indicates that targeting oxidative stress with elesclomol can improve the effectiveness of existing therapies for glioblastoma [114]. In animal models, treatment with elesclomol inhibited tumor growth and reduced the ability of tumor cells to spread along cerebrospinal fluid pathways [114].

### 3.10. Enzymatic Pathways and Oxidative Stress Inhibitors

NQO1 (NAD(P)H:quinone oxidoreductase 1) and GSTP1 (glutathione S-transferase pi 1) are cytoprotective enzymes that mitigate oxidative stress by limiting reactive oxygen species (ROS) accumulation [115,116,117,118,119]. NQO1 catalyzes the two-electron reduction in quinones, thereby preventing redox cycling and subsequent ROS formation, whereas GSTP1 promotes the conjugation of electrophilic compounds with glutathione, facilitating their detoxification [115,116,117,118,119]. Collectively, these enzymes attenuate ROS-mediated cellular damage; however, in glioblastoma (GBM), their upregulation confers a survival advantage, contributing to tumor resilience against oxidative stress [120,121]. In glioblastoma cells, these enzymes are often overexpressed, helping the cancer cells survive by managing oxidative stress levels [120]. A small molecule inhibitor called MNPC targets both NQO1 and GSTP1 [120]. By inhibiting these enzymes, MNPC increases oxidative stress in GBM cells, leading to higher levels of ROS, which is linked to cell damage and apoptosis [120]. When U87MG/EGFRvIII cells were treated with MNPC, researchers observed a dose-dependent increase in ROS levels [120]. This elevated oxidative stress was associated with cell death, indicating that MNPC effectively disrupts the redox balance in these cancer cells [120]. Treatment with MNPC also resulted in a decrease in the GSH to GSSG ratio, suggesting that the cells are under increased oxidative stress, as GSH is a major antioxidant that helps neutralize ROS [120]. MNPC treatment led to the activation of caspase 3, an important protein involved in the apoptosis pathway [120]. When NQO1 and GSTP1 were knocked down in the cells, there was a significant increase in ROS levels and cell death, confirming that these enzymes play a crucial role in managing oxidative stress in GBM cells [120].

Reyes-Soto et al. found that S-allyl-cysteine (SAC) decreases cell viability in glioblastoma cell lines RG2 and C6 in a concentration-dependent manner, suggesting that SAC may induce oxidative stress, contributing to its antiproliferative effects [122]. When SAC is combined with temozolomide, the study observed enhanced effects on oxidative stress markers, augmenting the lipoperoxidative effect of TMZ, which may lead to increased oxidative damage in the cancer cells [122]. Moreover, SAC reduces the antioxidant resistance of the glioblastoma cells by decreasing the GSH to GSSG ratio, indicating that SAC may compromise the cells’ ability to counteract oxidative stress [122]. While SAC alone does not affect Nrf2/ARE binding activity, the combination of SAC and TMZ decreases this activity, indicating a potential mechanism through which SAC enhances oxidative stress and contributes to the cytotoxic effects against glioblastoma cells [122].

Bufotalin treatment (BT) leads to significant oxidative stress in glioblastoma cells, evidenced by the generation of reactive oxygen species [123]. This increase in ROS levels is associated with cellular damage and apoptosis, indicating that BT effectively induces oxidative stress in GBM cells [123]. Alongside the increase in ROS, BT causes mitochondrial dysfunction, which can lead to increased oxidative stress and subsequent cell death [123]. The oxidative stress induced by BT is linked to the activation of apoptotic pathways, with the bursts of ROS contributing to the apoptosis of GBM cells [123]. The ITGB4/FAK/ERK signaling pathway is involved in the apoptosis induced by BT [123]. In addition, the downregulation of integrin β4 following BT suggests that this pathway may be influenced by the oxidative stress generated, further linking oxidative stress to the mechanism of action of BT in GBM [123].

### 3.11. Regulation of Oxidative Stress by Transcription Factors

C/EBPβ is a crucial transcription factor that responds to ROS and regulates the expression of NQO1 and GSTP1, which help neutralize ROS in glioblastoma cells [124]. This regulation is essential for the proliferation of brain tumors [124]. Overexpression of C/EBPβ leads to a selective decrease in ROS levels in EGFR-overexpressed U87MG cells [124]. This reduction in ROS is linked to the upregulation of NQO1 and GSTP1, which are responsible for combating oxidative stress [124]. Conversely, knocking down C/EBPβ results in elevated ROS levels, which negatively impacts cell proliferation [124]. This indicates that C/EBPβ plays a protective role against oxidative stress by promoting the expression of antioxidative enzymes [124]. C/EBPβ mediates brain tumor growth in vivo, correlating with the expression of NQO1 and GSTP1 and the levels of ROS [124]. Moreover, C/EBPβ upregulated in EGFR-overexpressed GBM cells is inversely correlated with the survival rates of glioblastoma patients [124]. It can be concluded that targeting C/EBPβ could be a potential therapeutic strategy to manage oxidative stress in this tumor [124].

### 3.12. Other ROS-Based Therapeutic Strategies

Cancer cells exploit redox signaling to encourage tumor growth and spread, leading to a reliance on antioxidant systems to manage oxidative stress and prevent cell death [125]. GBM cells depend heavily on their antioxidant systems to maintain a balanced redox state, which is crucial for avoiding excessive oxidative stress that can lead to cell damage and death [125]. Understanding these antioxidant networks in GBM can guide therapeutic strategies by targeting the specific redox states and antioxidant capacities of different GBM phenotypes, potentially leading to more effective treatments [125]. The study by Yang et al. identifies three distinct transcriptional co-expression networks (clusters C1, C2, and C3) based on their antioxidant capacities [125]. Cluster C1 exhibits strong antioxidant properties, while Cluster C2 displays a unique inflammatory trait and is associated with a higher level of oxidative stress, correlating with the aggressive mesenchymal subtype of GBM [125]. Cluster C3 has the weakest antioxidant capacity, suggesting a higher vulnerability to oxidative stress [125]. Patients with higher gene set variation analysis scores in the C2 cluster, linked to increased oxidative stress, showed poorer overall and progression-free survival outcomes, indicating that oxidative stress levels and antioxidant capacity can influence patient prognosis in GBM [125].

POLR2J expression is linked to the regulation of oxidative stress in glioblastoma cells [126]. High levels of POLR2J contribute to the malignancy of GBM by influencing oxidative stress pathways, which are crucial for cancer cell survival and proliferation [126]. When POLR2J is silenced, there is an activation of the unfolded protein response, indicating that the cells are experiencing stress, which can lead to increased apoptosis in GBM cells [126]. Silencing POLR2J significantly enhances the anti-glioblastoma activity of vorinostat, partly due to the suppression of cell proliferation and the induction of apoptosis influenced by oxidative stress levels [126]. The expression levels of POLR2J and its co-expressed genes can predict outcomes in GBM patients, suggesting that oxidative stress, regulated by POLR2J, plays a significant role in the aggressiveness of the tumor and the overall prognosis for patients [126].

Quéré et al. found that knocking out the ALDH1L2 gene in U251 glioblastoma cells led to an increase in oxidative stress [127]. The ROS levels in the knockout cells were significantly elevated compared to the wild-type cells in monolayer conditions [127]. This increase in ROS was linked to a reduction in total cellular NADPH levels in the knockout cells [127]. Even though the glutathione content remained stable, the cells’ ability to manage oxidative stress was compromised due to the knockout of ALDH1L2 [127]. The findings suggest that the loss of ALDH1L2 not only affects the production of NADPH but also leads to an accumulation of ROS, which can negatively impact cell health and function [127]. This highlights the importance of ALDH1L2 in maintaining redox balance and protecting glioblastoma stem cells from oxidative stress [127].

Glioblastoma exhibit high levels of reactive oxygen species due to their rapid growth, which contributes to their malignancy and resistance to temozolomide [98]. Standard treatment with radiotherapy combined with TMZ (RT/TMZ) reduces ROS levels, potentially limiting the therapy’s effectiveness [98]. Piperlongumine (PL) can enhance the efficacy of RT/TMZ by restoring ROS levels diminished by the therapy by depleting glutathione, leading to increased ROS accumulation and promoting cell death in GBM cells [98]. PL reprograms the expression of genes involved in ROS generation and scavenging, maintaining a balance that favors oxidative stress, thereby enhancing the therapeutic effects of RT/TMZ [98]. This combination could improve treatment outcomes by converting a “cold” tumor microenvironment into a “hot” one, more conducive to immune responses [98].

Photodynamic therapy (PDT) is a treatment that uses light-activated drugs to produce reactive oxygen species that can kill cancer cells, targeting tumor cells while minimizing damage to surrounding healthy tissue [128]. When the photosensitizer is activated by light, it generates singlet oxygen and other ROS, which can oxidize vital cellular components, leading to cell death [128]. However, nitric oxide produced by iNOS (inducible nitric oxide synthase) can contribute to resistance against PDT [128]. When glioblastoma cells are exposed to PDT, they can upregulate iNOS, leading to increased NO levels, which can protect the cells from oxidative damage caused by ROS [128]. Surviving glioblastoma cells that have been exposed to PDT and have high levels of NO tend to become more aggressive, showing increased proliferation, migration, and invasion capabilities [128]. Inhibitors of iNOS or NO scavengers can enhance the effectiveness of PDT by reducing the protective effects of NO [128].

## 4. Dietary and Pharmacological Antioxidant Compounds in Glioblastoma

Antioxidants, through their ability to modulate ROS levels, have garnered attention for their potential to enhance treatment efficacy or, conversely, to promote tumor survival depending on their context and concentration [64,66,125,129,130,131].

### 4.1. Glutathione

Glutathione (GSH) is a critical antioxidant in mammalian cells, playing a vital role in protecting against oxidative stress and maintaining cellular health [132,133]. This tripeptide is composed of glutamic acid, glycine, and cysteine [134]. GSH is involved in various cellular processes, including detoxification, redox signaling, and protein folding, and it serves as the body’s first line of defense against oxidative stress [132,135]. Its importance extends to its role in various health conditions, including cancer, diabetes, and neurodegenerative diseases [132]. GSH is capable of preventing damage to important cellular components caused by reactive oxygen species, free radicals, peroxides, lipid peroxides, and heavy metals [132,134].

In glioblastoma cells, glutathione plays a critical role in managing oxidative stress by neutralizing ROS [136]. GBM cells, particularly those with EGFR (epidermal growth factor receptor) overexpression, rely on GSH to survive the high levels of ROS in their environment [136]. The combination of auranofin, a thioredoxin reductase inhibitor, with L-BSO (L-buthionine-sulfoximine), which inhibits GSH synthesis, can significantly deplete intracellular GSH levels in GBM cells [136]. This depletion is associated with increased cytotoxicity, suggesting that reducing GSH levels can enhance the effectiveness of pro-oxidant treatments in these cancer cells [136]. The combination of auranofin and L-BSO led to synergistic cytotoxic effects across different GBM cell lines, regardless of their EGFR expression status, indicating that targeting GSH synthesis can be a viable strategy to increase the sensitivity of GBM cells to oxidative stress [136]. The extent of GSH depletion and the resulting cytotoxic effects can vary among different GBM cell lines, with U87/EGFRvIII cells exhibiting the most significant increase in ROS and the most pronounced cytotoxic response, suggesting that the presence of EGFR mutations may influence the effectiveness of GSH-targeting strategies [136].

The study by Franco et al. highlights the intricate relationship between GSH levels and tumor aggressiveness in astrocytomas [137]. Higher GS (glutathione synthetase) expression in the mesenchymal subtype of GBM suggests a reliance on increased GSH production for survival [137]. As astrocytomas increase in malignancy, GSH levels rise, potentially due to the downregulation of GLUD1 and GPT2, leading to increased glutamate availability for GSH synthesis [137]. Interestingly, in lower-grade astrocytomas with IDH1 mutations, higher GLUD1 and GPT2 expression correlates with lower GSH levels, potentially increasing sensitivity to oxidative stress and treatments like radiation therapy [137]. In the mesenchymal subtype, the downregulation of both genes and proteins (GLUD1 and GPT2) increases the source of glutamate for GSH synthesis and enhances tumor cell fitness due to increased antioxidative capacity [137]. In contrast, in lower-grade astrocytoma, mainly in those harboring the IDH1 mutation, the gene expression profile indicates that tumor cells might be sensitized to oxidative stress due to reduced GSH synthesis [137].

Cranial irradiation at 20 Gy significantly reduces GSH levels in the brain microenvironment, indicating its consumption to counteract radiation-induced oxidative stress [138]. This GSH reduction correlates with increased tumor aggressiveness, as lower antioxidant levels may foster a more permissive environment for tumor growth and recurrence, potentially due to oxidative stress promoting tumor cell proliferation and migration [138]. Metabolic changes post-radiation involve not only decreased GSH but also increased ATP and GTP, contributing to the aggressive behavior of GBM cells in the irradiated brain [138]. These findings suggest that managing GSH levels post-radiation could be a potential strategy to mitigate the adverse effects of RT on tumor recurrence [138]. Supplementing antioxidants like GSH after radiation therapy might improve outcomes for GBM patients [138].

TERT (telomerase reverse transcriptase), essential for GBM proliferation, upregulates the GSH pool by influencing FOXO1, which in turn increases GCLC (glutamate-cysteine ligase) expression, leading to GSH synthesis [139]. Inhibiting GCLC reduces GSH synthesis and impacts GBM cell clonogenicity, though it doesn’t directly cause cell death [139]. Interestingly, GBM cells exhibit compensatory mechanisms when GCLC is inhibited, increasing the metabolism of glutamine to glutamate and pyrimidine nucleotides, indicating metabolic plasticity [139]. Combining GCLC inhibition with GLS and CAD inhibitors shows promise as a synergistically lethal treatment for GBM cells, and in vivo experiments support the potential of targeting GSH metabolism in GBM therapy [139].

The study by Zhu et al. focused on the role of glutathione reductase (GSR) in drug resistance among glioblastoma cells, particularly in relation to temozolomide [140]. It was found that TMZ-resistant glioma cells had lower levels of reactive oxygen species (ROS) and higher levels of total antioxidant capacity and glutathione (GSH) compared to sensitive cells [140]. This suggests that resistant cells have developed a strong antioxidant defense to evade drug-induced damage [140]. GSR, a key enzyme in the GSH redox cycle, was expressed at higher levels in TMZ-resistant cells [140]. Silencing GSR in these resistant cells made them more sensitive to TMZ and cisplatin, indicating that GSR plays a crucial role in mediating drug resistance [140]. Conversely, overexpressing GSR in sensitive cells led to increased resistance to chemotherapy [140]. The study also highlighted that GSR helps maintain redox homeostasis, which is essential for cell survival under oxidative stress [140]. When GSR was knocked down, the resistant cells showed increased ROS levels, suggesting that GSR helps protect these cells from oxidative damage [140]. In vivo experiments using xenograft models demonstrated that knocking down GSR significantly reduced tumor growth and enhanced the effectiveness of TMZ treatment [140]. This indicates that targeting GSR could be a promising strategy to improve treatment outcomes for GBM patients [140]. The analysis of GSR expression in patient samples revealed that high levels of GSR were associated with shorter progress-free survival (PFS) in GBM patients [140]. Specifically, patients with high GSR expression had a median PFS of 11 months, compared to 14 months for those with low GSR expression [140]. This suggests that GSR could serve as a potential biomarker for predicting treatment response in GBM [140]. Overall, the findings suggest that GSR is a significant mediator of drug resistance in GBM cells and targeting it may enhance the efficacy of current chemotherapeutic agents, providing a potential new avenue for improving GBM treatment [140].

Guo et al. investigated the expression of GPX2 (glutathione peroxidase 2) in glioblastoma and its potential role as a prognostic indicator [141]. It was found that GPX2 expression did not show significant differences between normal brain tissues and GBM tissues [141]. Despite the lack of significant differences in expression levels, the study revealed that higher GPX2 expression was correlated with poorer overall survival in GBM patients [141]. Specifically, the analysis indicated a significant association between elevated GPX2 levels and reduced survival rates [141]. The research also explored the methylation status of GPX2 in GBM, finding no significant differences compared to normal tissues [141]. This suggests that GPX2’s role in GBM may not be primarily influenced by methylation changes [141]. The study identified coexpressed genes associated with GPX2 and constructed a protein–protein interaction (PPI) network [141]. The analysis highlighted the chemokine-signaling pathway as a significant pathway related to GPX2, indicating its potential involvement in GBM progression [141]. Overall, GPX2 appears to be a candidate proto-oncogene in GBM, and its expression levels could help predict patient outcomes, making it a valuable focus for future studies in cancer treatment strategies [141].

The study by Zheng et al. describes a multifunctional nanoplatform designed to respond to the high concentrations of glutathione found in the tumor microenvironment [142]. When the nanoparticles are exposed to GSH, they rapidly disintegrate, releasing manganese ions and doxorubicin, which directly targets and kills glioma cells [142]. The released doxorubicin also catalyzes the release of mitochondrial DNA, triggering immunogenic cell death, which can stimulate an immune response against the tumor [142]. Furthermore, both the released doxorubicin and the manganese ions activate the cGAS-STING pathway, which plays a crucial role in reshaping the immunosuppressive TME (tumor microenvironment) and enhancing the overall effectiveness of chemotherapy for glioma [142]. This GSH-responsive mechanism significantly contributes to the nanoplatform’s ability to inhibit tumor growth [142]. By leveraging the high GSH levels in the TME, the platform effectively enhances the delivery and efficacy of chemotherapy, offering a promising strategy for glioma treatment [142].

Feng et al. introduced a virus-inspired biodegradable tetrasulfide-bridged mesoporous organosilica (vMSTI) nanosystem designed to co-load TMZ and indocyanine green to facilitate fluorescence imaging-guided sonodynamic chemotherapy while targeting GSH depletion [143]. The vMSTI nanosystem accumulates in tumor cells and promotes GSH depletion to enhance the cytotoxic effects of TMZ, potentially overcoming GBM cell resistance mechanisms [143]. The combination of GSH depletion and TMZ delivery via vMSTI aims to improve therapeutic outcomes in GBM by addressing inadequate drug delivery and the protective role of GSH in tumor cells [143]. The findings suggest that targeting GSH levels in GBM treatment could be a promising strategy, with the vMSTI system potentially enhancing chemotherapy efficacy and improving patient outcomes [143].

### 4.2. SOD

Superoxide dismutases (SODs) are responsible for managing oxidative stress by catalyzing the dismutation of superoxide radicals into oxygen and hydrogen peroxide [144]. In glioblastoma, SODs are implicated in various mechanisms that contribute to tumor progression, therapy resistance, and immune modulation [129,144].

High levels of oxidative stress-responsive genes, including SOD3 (Superoxide Dismutase 3), correlate with poor prognosis in glioblastoma patients [75]. Knockdown of SOD3 decreases the M2-like pro-tumoral transformation of macrophages both in vitro and in vivo, suggesting SOD3’s potential role in regulating macrophage M2-like pro-tumoral transformation, which is associated with immunosuppression and tumor progression [75]. The presence of M2-like macrophages is linked to a more aggressive tumor phenotype, and targeting SOD3 could potentially modulate the immune environment to favor anti-tumor responses [75]. This finding indicates that SOD3 could be a novel target for developing therapeutic strategies aimed at modulating the immune response within the tumor microenvironment in glioblastoma [75].

SOD2 (Superoxide Dismutase 2) is highly upregulated in the mesenchymal subtype of glioblastoma [145]. Temozolomide-resistant GBM cells activate the CYBB/Nrf2/SOD2 axis, contributing to their resilience against erastin-mediated ferroptosis [145]. CYBB interacts with Nrf2, which regulates SOD2 transcription, and the compensatory antioxidant activity of SOD2 is essential for protecting TMZ-resistant cells from high reactive oxygen species and attenuating ferroptosis [145]. An animal study further highlighted the protective function of SOD2, showing that it mitigates erastin-triggered ferroptosis and enables tolerance to oxidative stress burden in mice harboring TMZ-resistant GBM cell xenografts [145].

### 4.3. CAT

Catalase (CAT), a pivotal enzyme, facilitates the decomposition of hydrogen peroxide into water and oxygen, thereby playing a critical role in shielding cells from oxidative damage [130,146]. The role of CAT in glioblastoma presents a complex and seemingly paradoxical picture that requires careful interpretation of the existing literature. Initial studies demonstrated that compared to normal brain tissue, brain tumor tissue exhibits considerably less H_2_O_2_ detoxification by CAT [130]. Specifically, catalase levels have been found to be decreased in the nucleus and mitochondria of brain tumor cells [147,148]. These findings suggest reduced CAT activity in specific subcellular compartments where oxidative damage could be particularly detrimental. However, functional studies reveal that when CAT is overexpressed in glioma cells, it leads to a more aggressive cancer phenotype [146]. This overexpression is linked to increased resistance to standard treatments like temozolomide and radiation therapy [146]. Clinically, higher levels of CAT expression were associated with poorer overall survival rates in patients with high-grade glioma [146].

These apparently conflicting results can be reconciled by considering several factors. First, CAT levels may differ depending on the subcellular compartment examined. Reductions observed in the nucleus and mitochondria do not necessarily indicate a decrease in total cellular CAT, as glioma cells may redistribute CAT to the cytoplasm or regulate its expression differently across compartments [147,148]. Second, variability in CAT expression may reflect tumor heterogeneity and disease progression. Distinct glioma subtypes, grades, or stages may exhibit unique expression patterns, with earlier studies reporting reduced CAT activity likely examining different tumor populations than those characterized by overexpression in aggressive phenotypes [130,146]. Finally, CAT overexpression in advanced or treatment-exposed gliomas may represent an adaptive resistance mechanism, enabling tumor cells to mitigate therapy-induced oxidative stress and enhance survival.

The functional significance of CAT overexpression is demonstrated by its impact on treatment efficacy. Catalase overexpression reduces the basal levels of hydrogen peroxide, a type of reactive oxygen species, which is crucial for the effectiveness of chemotherapy and radiotherapy [146]. This mechanism explains the observed treatment resistance in CAT-overexpressing tumors [146]. Importantly, pharmacological inhibition of CAT activity led to reduced proliferation of glioma cells derived from patient biopsies, suggesting that targeting CAT could be a potential therapeutic strategy to overcome resistance in glioblastoma [146]. This finding supports the clinical relevance of CAT as a therapeutic target, particularly in treatment-resistant cases where CAT overexpression may be driving aggressive behavior and poor outcomes.

### 4.4. Carotenoids

Carotenoids are naturally occurring dietary antioxidants with chemopreventive and potential chemotherapeutic properties for various cancers, including CNS tumors [149]. They exhibit chemopreventive effects by suppressing the harmful effects of free radicals that regulate cancer cell proliferation, cell cycle progression, invasion, inflammation, and angiogenesis, by regulating molecular events such as Akt/PI3K/mTOR, cyclin/CDK, PPAR (Peroxisome Proliferator-Activated Receptor), Wnt (Wingless-related integration site), VEGF (Vascular Endothelial Growth Factor), MMPs (Matrix Metalloproteinases), and NF-κB (nuclear factor kappa-light-chain-enhancer of activated B cells) signaling [150,151]. Carotenoids can also promote ROS production with prooxidant chemistry, aiding their chemotherapeutic potential [152]. Preclinical evidence suggests that carotenoids can target apoptotic processes to improve cancer management [153].

#### 4.4.1. Astaxanthin

Astaxanthin(AXT) is a natural carotenoid, primarily derived from microalgae and seafood, with strong antioxidant properties, which allows it to combat oxidative stress [154,155,156]. It has been shown to target reactive oxygen species and by modulating their levels, it may help in reducing oxidative stress within GBM cells, potentially leading to decreased tumor growth [154,156,157,158]. This characteristic makes it a promising candidate for further research and development in cancer therapies aimed at glioblastoma.

Siangcham et al. found that astaxanthin significantly decreases the migration and invasion of A172 human glioblastoma cells and shows no toxicity at concentrations up to 150 µM after 48 h of treatment [155]. This effect is observed as ATX reduces the expression of matrix metalloproteinase-2 and the activity of matrix metalloproteinase-9 [155]. Moreover, the effects of ATX on these proteins were dose-dependent, meaning higher concentrations of ATX led to greater reductions in MMP-2 and MMP-9 levels [155].

#### 4.4.2. Lycopene

Lycopene, a natural pigment found in various fruits and vegetables, particularly in tomatoes, watermelon, and pink grapefruit [159,160,161]. It is a carotenoid, which gives these foods their red and pink colors [160,161]. Lycopene is known for its antioxidant properties [159,160,161]. It increases levels of intracellular and mitochondrial reactive oxygen species in GBM8401 and T98G cells [162]. This increase in ROS is a critical factor in the mechanism by which lycopene exerts its cytotoxic effects on glioblastoma cells, with elevated ROS levels implicated in the activation of the ERK signaling pathway [162]. The oxidative stress resulting from increased ROS levels contributed to the upregulation of p53, a protein that is essential for inducing apoptosis and regulating the cell cycle [162]. By promoting oxidative stress, lycopene effectively inhibits cell proliferation and induces apoptosis in glioblastoma cells, making it a promising candidate for further research in cancer treatment [162].

Lycopene’s impact on glioblastoma includes a significant decrease in the viability of U118-MG glioblastoma cells, indicating its potential as an anticancer agent [163]. Lycopene stimulates apoptosis in glioblastoma cells, promoting programmed cell death [163]. Research also suggests that lycopene influences mitochondrial function, contributing to the apoptotic process in GBM [163]. The effects of lycopene on apoptosis and cell viability are dose-dependent, meaning that higher concentrations of lycopene lead to more significant reductions in cell viability and increased apoptosis, indicating that the dosage of lycopene is an important factor in its effectiveness [163].

In glioblastoma, lycopene’s activity shifts in a context-dependent manner, displaying pro-oxidant effects that selectively harm cancer cells. This paradox can be explained by several mechanisms. First, lycopene increases intracellular and mitochondrial reactive oxygen species specifically in glioblastoma cell lines such as GBM8401 and T98G, exploiting their altered redox balance and impaired antioxidant defenses [162]. Second, it disrupts mitochondrial electron transport, leading to excess electron leakage and superoxide generation, which activates apoptosis-related pathways including p53 upregulation and ERK signaling [162,163]. Third, this effect is dose-dependent, as higher concentrations of lycopene enhance apoptosis and reduce cell viability in U118-MG glioblastoma cells [163]. And finally, while inducing oxidative stress in cancer cells, lycopene preserves its antioxidant role in healthy cells, protecting them from damage [159,160,161]. Together, these findings suggest that lycopene’s biological activity is shaped by the cellular redox environment and mitochondrial function, allowing it to act as a selective pro-oxidant in glioblastoma while maintaining antioxidant protection in normal tissue.

#### 4.4.3. Crocetin

Crocetin is a naturally occurring carotenoid dicarboxylic acid derived from saffron (*Crocus sativus* L.) and *Gardenia jasminoides* Ellis [164]. It exhibits diverse pharmacological properties, including cardioprotective, neuroprotective, and anti-inflammatory effects [164]. Crocetin is considered one of the main antioxidant constituents of saffron [165]. It shows significant antitumor properties against glioblastoma in both laboratory and animal models [166]. Crocetin was effective in inhibiting the proliferation of four different GBM cell lines (U251, U87, U138, and U373) [166]. Treatment with the use of crocetin led to a decrease in mesenchymal markers and an increase in neuronal markers, suggesting that it may help in differentiating aggressive cancer cells into less harmful forms [166]. In animal models, crocetin was found to significantly inhibit tumor growth when compared to standard treatments like radiotherapy and temozolomide [166]. Moreover, the treatment with crocetin improved disease-free survival (DFS) and overall survival (OS) rates in the animal models [166].

Crocetin significantly reduces the viability, proliferation, and migration of U87 glioma cells, at concentrations ranging from 75–150 µM [167]. It achieves its effects by decreasing the levels of proteins such as Matrix Metallopeptidase 9 and Ras homolog family member A [167]. This treatment can block multiple points of the AKT signaling pathway, which is essential for cancer cell survival, leading to increased cell death and disruption of the cell cycle in glioma cells [167]. When combined with temozolomide, this combination therapy enhances the reduction in cancer cell growth and promotes cell death [167].

#### 4.4.4. Zeaxanthin

Zeaxanthin (Zea) is a carotenoid pigment belonging to the xanthophyll family, known for its significant health benefits due to its antioxidant and anti-inflammatory properties [168,169]. It is naturally found in various fruits and vegetables and plays a crucial role in human health, particularly in eye health, and potentially in cancer prevention and cardiovascular protection [168,169]. Zea demonstrates the ability to hinder tumor growth and angiogenesis effectively in an in vivo human GBM xenograft mouse model [170]. Zeaxanthin significantly impairs the proliferation, adhesion, migration, and invasion of human glioblastoma cell lines, specifically U87 and U251 [170]. It inhibits angiogenesis and induces apoptosis in GBM cells by increasing the expression of cleaved PARP and Caspase 3, which are markers of programmed cell death [170]. Moreover, it downregulates the activation of the VEGFR2 kinase pathway and disrupts multiple oncogenic signaling pathways [170].

### 4.5. Coenzyme Q10

Coenzyme Q10 (CoQ10) is a crucial component of the mitochondrial electron transport chain, facilitating the production of adenosine triphosphate, which is essential for cellular energy [171,172]. It exists in two forms: ubiquinone (oxidized) and ubiquinol (reduced), both of which contribute to its antioxidant properties by scavenging free radicals and regenerating other antioxidants like tocopherol [171,173]. As an antioxidant, CoQ10 protects cells from oxidative damage, which is linked to aging and various diseases [174]. When delivered via BPM31510, a lipid nanodispersion, CoQ10 has been shown to enhance the effects of radiation therapy in glioblastoma models [175]. This combination resulted in a significant extension of survival in rodent models, indicating a potential synergistic relationship between CoQ10 and radiation through the selective enhancement of oxidative stress within tumor cells [175]. Moreover, another research indicates that CoQ10 is capable of preserving the structural integrity of the glial fibrillary acidic protein network in astrocytes following radiation exposure [176]. This action may mitigate radiation-induced damage while simultaneously augmenting the therapeutic efficacy against glioma cells [176].

Coenzyme Q10 supplementation could be a valuable addition to standard glioblastoma treatment, as it enhances the effectiveness of temozolomide, as it makes the glioma cells more sensitive to TMZ, and reduces the invasiveness of glioma cells [177]. CoQ10 treatment leads to a reduction in ROS levels in the glioma cells, and effectively modulates oxidative stress in the cells [177]. Coenzyme Q10 and TMZ treatments reduce the ability of RC6 cells to invade, and the combination treatment was particularly effective [177].

CoQ10, particularly its oxidazed form-ubidecarenone, is being explored in a drug-lipid conjugate nanodispersion called BPM31510 to improve its bioavailability for cancer treatment [178]. While CoQ10 is known for its antioxidant properties, high concentrations can have a pro-oxidant effect in malignant cells, potentially leading to their death [178]. Glioblastoma multiforme relies on redox homeostasis to prevent apoptosis from reactive oxygen species, and research is being done to see if BPM31510 with chemoradiation can exploit this vulnerability [178]. Studies have indicated a favorable safety profile for BPM31510, with Vitamin K co-administration helping to mitigate coagulopathy [178]. CoQ10 acts as a sensitizer for cytotoxic therapies, and disarms GBM cells against further pro-oxidant injuries, being potentially useful in clinical practice for this fatal pathology [178].

In glioblastoma, the apparent contradiction in CoQ10’s mechanism is explained by its concentration-dependent and context-specific effects. CoQ10 exists in both ubiquinone and ubiquinol forms, which normally act as antioxidants by scavenging free radicals and regenerating tocopherol [171,173]. At higher therapeutic concentrations, however, CoQ10 can shift toward pro-oxidant activity in malignant cells, particularly when delivered through formulations such as BPM31510 [178]. This biphasic response is shaped by the cellular redox state: while normal cells maintain redox balance and benefit from CoQ10’s protective effects, glioblastoma cells already exist under elevated oxidative stress with weakened defenses, making them more vulnerable to CoQ10-induced disruption [178]. Evidence also shows that CoQ10 modulates baseline ROS in glioma cells, lowering stress adaptation and thereby enhancing the effectiveness of radiotherapy and temozolomide [177]. Importantly, this modulation sensitizes glioblastoma cells to treatment while protecting normal astrocytes, where CoQ10 preserves glial fibrillary acidic protein integrity against radiation damage [176]. Since CoQ10 is integral to the mitochondrial electron transport chain, its therapeutic doses may further disrupt cancer-specific metabolic patterns, creating dependencies that increase susceptibility to ROS-mediated therapies [171,172,178]. Thus, CoQ10 functions not as a simple antioxidant or pro-oxidant, but as a selective redox and metabolic modulator with distinct effects in healthy and malignant cells.

### 4.6. Curcumin

Curcumin, a compound derived from turmeric (*Curcuma longa*), has been widely investigated for its various therapeutic properties, including its anti-inflammatory, antioxidant, and anticarcinogenic effects [179,180,181]. Curcumin has demonstrated effectiveness against glioblastoma due to its ability to modulate key signaling pathways involved in GBM cell proliferation, apoptosis, cell cycle arrest, autophagy, and oxidative stress [181,182,183]. It targets pathways such as Rb, p53, MAPK, PI3K/Akt, JAK/STAT, Shh, and NF-κB, which are often dysregulated in GBM [181,182,183]. Curcumin appears to induce DNA damage and apoptosis in glioma cells, potentially through a ROS-dependent mechanism [184]. The study by Seyithanoğlu et al. showed that higher doses of curcumin resulted in greater cellular death of cancer cells compared to normal cells, along with increased ROS generation in cancer cells [184]. Curcumin induced DNA damage in a dose-dependent manner, and higher concentrations led to greater apoptosis in cancer cells [184].

Curcumin demonstrates significant potential in targeting Glioblastoma Stem Cells [185]. It reduces GSCs viability in a dose-dependent manner, with an IC50 around 25 μM [185]. Even at lower concentrations, it inhibits GSCs proliferation, sphere-forming ability, and colony-forming potential [185]. The anticancer effects of curcumin are mediated through ROS induction [185]. It also impacts signaling pathways by activating MAPK and inhibiting the STAT3 pathway, which is crucial for GSC survival [185].

Curcumin enhances the efficacy of radiation therapy, particularly in neutron-irradiated cell lines [181,182,183]. When combined with radiation, it increases apoptosis and raises levels of reactive oxygen species [183]. This combination also significantly inhibits cell invasion compared to treatments with curcumin alone [183]. The findings suggest that curcumin could have clinical utility in combination cancer treatments, especially with high-LET radiation therapies [183].

Combining curcumin and radiation significantly reduces cell viability in both U87 and T98 cell lines, leading to a higher arrest of cells at the G2/M phase of the cell cycle [186]. Curcumin, combined with temozolomide, results in increased tumor cell death, suggesting that curcumin could enhance the effectiveness of existing treatments for glioblastoma [186]. Low doses of curcumin combined with radiation therapy exhibit a strong synergistic anti-proliferative effect on glioblastoma cells in vitro, representing a promising strategy for glioblastoma treatment [186].

Glioblastoma’s resistance to temozolomide is a significant challenge, leading to low patient survival rates [187]. Combining curcumin and polydatin enhances the cytotoxic effects of TMZ on glioblastoma cells, especially those resistant to the drug, improving cell viability and altering cell morphology [187]. This combination influences cell cycle progression, apoptosis, and autophagy, reducing cell proliferation and increasing differentiation of glioblastoma cells [187]. CUR and PLD modulate the expression of proteins related to cell survival and apoptosis, favoring tumor cell death and reduced invasiveness [187]. It can be concluded that the synergistic combination of CUR and PLD significantly enhances the efficacy of TMZ against drug-resistant GBL cells [187].

Curcumin reveals a protective effect against homocysteine-induced damage in U373 glioblastoma cells, mediated through the Nrf2/HO-1 signaling pathway and TIGAR expression [188]. Curcumin partially enhances cell viability and attenuates intracellular ROS formation in Hcy-treated cells [188]. It also moderates Hcy-induced oxidative damage via HO-1 expression by activating Nrf2 expression, with TIGAR contributing to the protective effects [188].

Curcumin’s impact on glioblastoma cell viability and autophagy is significant [189]. It effectively induces cell death in glioblastoma cells, with 10 μM curcumin reducing A172 cell viability substantially (after 24 h it dropped to 65%, and after 48 h it further decreased to 52% compared to control cells) [189]. It’s important to note that curcumin can induce autophagy, and this curcumin-induced autophagy enhances cell death [189]. However, it appears that the level of autophagy beforehand can change things, as increasing the basal level of autophagy can decrease curcumin’s effectiveness, suggesting that an already high level of autophagy can be counterproductive [189].

The study by Su et al. identified a total of 16 target genes of curcumin that are relevant to glioblastoma, among which three—ENO1, MMP2 and PRKD2—were found to significantly impact the prognosis of GBM patients (*p* < 0.05) [190]. In vitro experiments, including migration and invasion assays, confirm that curcumin effectively inhibits the migration and invasion abilities of U251 GBM cells, while flow cytometry indicates that curcumin promotes apoptosis [190].

Luo et al. investigated the anticancer effects of four curcumin analogues on human glioma cells [191]. All four analogues (curcumin, bisdemethoxycurcumin, demethoxycurcumin, and dimethoxycurcumin) increased apoptosis and caused cell cycle arrest in the glioma cells [191]. Dimethoxycurcumin was particularly effective, promoting apoptosis, increasing ROS production, and suppressing cell viability and migration [191]. The study highlighted that dimethoxycurcumin promotes apoptosis and autophagy by increasing the expression of p-AKT, p-ERK, LC3B-II and p62, while reducing p-mTOR and BCL-2 [191]. This suggests that dimethoxycurcumin induces both apoptosis and autophagy in glioma cells. The authors concluded that the number of methoxy groups in curcumin analogues correlates with their anticancer activity [191].

The development of targeted drug delivery systems for curcumin shows great promise in GBM treatment. A novel system using curcumin combined with MPEG-PLA and Fa-PEG-PLA enhances targeting of GBM cells [192]. This curcumin/Fa-PEG-PLA formulation significantly outperforms free curcumin and Cur/MPEG-PLA in suppressing GL261 cell growth and promoting apoptosis [192]. Furthermore, in vivo experiments show significant tumor growth repression with curcumin/Fa-PEG-PLA in both subcutaneous and intracranial models, attributed to suppressed angiogenesis and facilitated apoptosis [192]. In conlusion, curcumin-loaded nanoparticles can be used as an effective delivery system to overcome the challenges of drug delivery to the brain, providing a new approach to glioblastoma therapy [192].

Curcumin-encapsulated dendrimers significantly reduced the viability of glioblastoma cell lines compared to non-cancerous control cells [193]. The encapsulation of curcumin within dendrimers enhances its efficacy against cancer cells [193]. Conversely, unencapsulated curcumin demonstrates limited effectiveness in diminishing cell viability, underscoring the importance of the delivery method for its therapeutic potential [193].The use of surface-modified PAMAM dendrimers to deliver curcumin can provide a promising new approach for treating glioblastoma, as it specifically targets cancer cells while minimizing effects on normal cells [193].

### 4.7. Flavonoids

Flavonoids are a diverse group of polyphenolic compounds naturally found in plants, including fruits, vegetables, grains, and beverages like tea and wine [194,195]. Recognized for their antioxidant, anti-inflammatory, and anticancer properties, they are categorized into subclasses like flavones, flavonols, flavanones, isoflavones, flavandiols, and anthocyanidins, each with unique structures and activities [194,195,196]. Flavonoids exhibit anti-inflammatory, antithrombogenic, antidiabetic, anticancer, and neuroprotective activities through different mechanisms of action [194,195,197,198]. Due to variations in their chemical structure, bioavailability and metabolism, different flavonoid compounds exert a range of biological effects, including anti-inflammatory and antioxidative stress effects [195,197,199].

#### 4.7.1. Quercetin

The study by Kusaczuk et al. highlights the potential of quercetin (QCT) and kaempferol (KMF) as therapeutic agents in glioblastoma treatment [200]. Both QCT and KMF significantly reduced the viability of glioblastoma cells, indicating their potential as cytostatic agents [200]. The study demonstrated that QCT and KMF induced apoptotic cell death in T98G cells, evidenced by increased activity of caspases and overexpression of cleaved PARP (Poly (ADP-ribose) polymerase) [200]. The treatment with both flavonoids led to the activation of stress responses in the GBM cells, specifically oxidative stress, indicated by increased ROS generation, changes in SOD expression, and overexpression of stress-related proteins [200]. The antitumor effects of QCT and KMF were confirmed in vivo, with reduced growth of tumor xenografts observed [200]. Both compounds were found to comply with Lipinski’s Rule of 5, suggesting they have favorable drug-like properties and are suitable candidates for central nervous system drug development [200].

The viability reduction in glioblastoma cells in both laboratory and animal models is dose-dependent, with higher concentrations leading to greater decreases in cell survival [201]. Quercetin markedly suppresses the migration and invasion of glioblastoma cells, as demonstrated through wound healing and transwell assays [201]. Moreover, it inhibits the EMT (Epithelial–Mesenchymal Transition) process, increasing the expression of E-cadherin (an epithelial marker) while decreasing N-cadherin and vimentin (mesenchymal markers) [201]. Quercetin exerts its effects by suppressing the GSK3β/β-catenin/ZEB1 signaling pathway, which plays a significant role in promoting EMT and cancer cell invasion [201]. In animal models, QCT treatment resulted in reduced tumor volume and weight, along with lower expression of Ki67, supporting its potential as a therapeutic agent against glioblastoma [201].

Quercetin treatment significantly reduces the viability of glioblastoma cell lines U87MG and U373MG, while preserving a significant portion of normal astrocyte cells, suggesting a selective effect on cancer cells [202]. Furthermore, QCT induces apoptosis in glioblastoma cells, without affecting the Akt or mitogen-activated protein kinases typically associated with cell proliferation [202]. Quercetin treatment also leads to a decrease in the release of IL-6 and the phosphorylation of STAT3, suggesting interference with signaling pathways that promote tumor growth and survival [202]. The study demonstrates that quercetin suppresses gene expression, protein expression, and the half-life of the synthesized Axl protein, a receptor tyrosine kinase that plays a role in cancer progression [202]. The importance of Axl in the apoptotic effect of quercetin was confirmed using shRNA to knock down Axl expression, further establishing Axl as a significant target for quercetin’s anticancer action in glioblastoma cells [202]. Overall, these findings suggest that quercetin has potential as an anticancer agent for glioblastoma treatment, potentially improving the cancer microenvironment and offering a new therapeutic strategy for managing glioblastoma by targeting the Axl/IL-6/STAT3 signaling pathway [202].

Baba et al. confirmed quercetin’s effects on reducing the viability and migration of glioma cells, an effect observed in both naïve cells and those transfected with Rac1 and p66Shc, indicating broad effectiveness [203]. The reduction in cell viability and migration is dependent on ROS levels, with quercetin lowering ROS production, which mitigates oxidative stress associated with gliomas [203]. Quercetin inhibits the expression of Rac1 and p66Shc, key proteins involved in tumor-associated inflammation and ROS generation [203]. This inhibition is significant as it suggests a mechanism through which quercetin can exert its anticancer effects [203]. Molecular docking simulations demonstrated quercetin’s strong binding affinity to Rac1, blocking the GTP-binding site and preventing its activation, highlighting a specific molecular target for quercetin’s action [203].

Wang et al. demonstrated significant effects of QCT on glioblastoma cells, including the inhibition of cell proliferation, promotion of apoptosis, and reduction in tumor growth in animal models [204]. In addition, quercetin administration has also been shown to improve locomotor activity and enhance the survival rate of mice with GBM [204]. The proposed mechanism of action involves the inhibition of glycolytic metabolism in tumor tissues, disrupting the energy supply crucial for cancer cell growth and survival [204].

The study by Pourmasoumi et al. focused on enhancing glioblastoma multiforme treatment by co-delivering temozolomide and quercetin using folic acid-conjugated exosomes [205]. This co-delivery system has the potential to overcome the resistance that GBM cells often develop against TMZ, a major hurdle in effective treatment [205]. The use of FA-conjugated exosomes is particularly important for improving drug delivery across the BBB, potentially enhancing the concentration of therapeutic agents in the tumor site [205].

Bhandarkar et al. introduced a novel drug delivery system using natural human platelets as carriers for quercetin. The platelets were selected due to their ability to effectively encapsulate drugs, utilizing their open canalicular system for drug uptake [206]. The research demonstrated a high encapsulation efficiency of 93.96 ± 0.12% for quercetin within the platelets, indicating that a significant amount of the drug can be loaded into the carrier [206]. The maximum drug release from the QCT-platelet system was 76.26 ± 0.13% within 24 h at a pH of 5.5, simulating the acidic environment of tumors, suggesting effective drug release in the tumor microenvironment [206]. Cytotoxic effects of the QCT-platelets were evaluated on the U373-MG human astrocytoma glioblastoma cell line, and after 48 h of treatment, the cell viability was significantly reduced to 14.52 ± 1.53%, indicating that the QCT-platelet system effectively kills glioblastoma cells [206]. Concluding, quercetin-loaded platelets can serve as effective carriers for therapeutic agents, providing a promising targeted therapy for glioblastoma, which may enhance treatment efficacy and overcome the limitations associated with current therapies [206].

Fisetin and quercetin protect non-cancerous cells while effectively eliminating transformed cancer cells in both 2D cultures and 3D tumoroids, indicating their potential as therapeutic agents in treating GBM [207]. Fisetin decreases cytosolic acetylated high mobility group box 1 (acHMGB1) and increases the abundance of transcription factor EB in microglia, but not in GBM cells, suggesting a selective action of fisetin in the tumor microenvironment [207]. Fisetin is a potent modulator of the Nrf2-KEAP1 complex, crucial for cellular responses to oxidative stress [207]. Molecular modeling indicates that fisetin has more interaction sites and stronger binding affinities with the Nrf2-KEAP1 complex compared to quercetin, highlighting fisetin’s potential as a more effective therapeutic agent in managing oxidative stress in GBM [207].

#### 4.7.2. Fisetin

Fisetin exhibits cytotoxic effects on LN229 glioblastoma cells, leading to increased apoptosis [208]. It induces DNA damage in glioblastoma cells within a dose range of 40–80 μM, measured using the alkaline comet assay and the γH2AX assay [208]. Fisetin triggers a DNA damage response, as evidenced by the activation of the p53 protein, suggesting that fisetin not only damages DNA but also activates cellular mechanisms to respond to this damage [208]. It enhances the genotoxic effects of methyl methanesulfonate, a known DNA-damaging agent, likely due to fisetin’s ability to inhibit DNA repair processes, making cancer cells more susceptible to damage [208]. When administered alongside temozolomide, a first-line treatment for glioblastoma, fisetin significantly increased cell death and reduced the number of therapy-induced senescent cells, suggesting that fisetin may have senolytic activity, which could help eliminate cells that have stopped dividing but are not dead [208]. These findings suggest that high-dose fisetin has a genotoxic potential and could enhance the effects of anticancer drugs like temozolomide [208].

#### 4.7.3. Rutin

Treatment with rutin and quercetin significantly inhibited the proliferation and migration of rat C6 glioma cells [209]. This suggests that these flavonoids can directly affect glioma cell behavior, potentially slowing tumor growth [209]. The flavonoid treatment induced microglia chemotaxis, which is the movement of microglia towards the glioma cells [209]. This was associated with an increase in tumor necrosis factor levels and a decrease in Interleukin 10 levels, indicating a shift in the inflammatory profile of microglia [209]. The study found that rutin and quercetin treatment led to changes in the expression of various chemokines (CCL2, CCL5, CX3CL1) and growth factors (Heparin Binding Growth Factor, Insulin-like growth factor, Glial cell-derived neurotrophic factor) [209]. These changes are crucial for understanding how these flavonoids modulate the tumor microenvironment [209]. In human U251 and TG1 glioblastoma cells, rutin and quercetin reduced the expression of mRNA for IL-6 and IL-10 while increasing TNF expression [209]. This indicates a shift towards a more inflammatory immune response, which may be beneficial in combating glioma [209]. When U251 cells treated with flavonoids were xenotransplanted into rat brains, there was a reduction in tumorigenesis [209]. The treatment also influenced the microglial and astrocytic response in the tumor microenvironment, leading to a less favorable profile for glioma growth [209].

Rutin treatment (30−50 μM) significantly decrease the viability of human GL15-GBM cells, indicating a potent anti-glioma effect [210]. Interestingly, it did not affect the viability of human C20 microglia, suggesting that it can target GBM cells effectively while sparing microglial cells [210]. The findings also showed that rutin has antiproliferative and proapoptotic effects on glioblastoma cells [210]. 30 μM significantly reduced the expression of miRNA-125b in GBM cells and their secretome, which is crucial as miRNA-125b is associated with various cancer-related processes, including proliferation and apoptosis [210]. Rutin treatment also led to a significant reduction in STAT3 expression in GBM cells [210]. When microglia were exposed to the conditioned medium from GBM cells treated with rutin, they exhibited a reactive morphology [210]. This change was associated with a decrease in the expression of pro-inflammatory cytokines such as IL-6 and TNF, as well as reduced STAT3 expression in microglia [210]. This suggests that rutin not only affects GBM cells but also modulates the inflammatory profile of microglia, potentially shifting them towards a more anti-tumor phenotype [210].

Hydrolized rutin (HR) demonstrates a strong ability to inhibit the proliferation of glioblastoma cells, specifically using the U251 glioblastoma cell line, where HR effectively reduce cell growth, indicating its potential as a therapeutic agent against glioblastoma [211]. The compound was found to inhibit the cell cycle, which is crucial for controlling tumor growth, through a p53-independent pathway, suggesting that HR may operate through mechanisms distinct from traditional tumor suppressor pathways [211]. In animal models of glioblastoma, treatment with HR led to a significant decrease in tumor growth and aggression, evidenced by lower rates of mitosis and necrosis, which are indicators of tumor malignancy [211]. Histological analyses showed that HR interfered with aggressive tumor behavior and reduced glioma grade, suggesting that HR not only slows down tumor growth but may also improve the overall histological characteristics of the tumor [211]. HR acts without causing genotoxicity, which is a significant advantage as it suggests a safer profile for potential therapeutic use [211].

The combination of rutin with Temozolomide, referred to as PLArutin, resulted in rapid inhibition of glioblastoma cell growth, indicating that rutin is effective in quickly reducing the viability of these cancer cells, which is crucial for aggressive tumor treatment [212]. The study by Erçelik et al. utilized various assays to evaluate the impact of rutin on GBM cells, including cell viability tests and assessments of migration capacity [212]. The results indicated that all polymeric nanofiber webs, including those with rutin, promoted apoptotic cell death and suppressed cell migration, which are critical factors in cancer progression [212].

#### 4.7.4. Apigenin

Apigenin has demonstrated significant potential as an anticancer agent, notably in reducing the viability of glioblastoma cells (U1242 MG and U87 MG) in a dose- and time-dependent manner [213]. As the concentration increased, the number of viable cells decreased, indicating its effectiveness in inhibiting cell growth [213]. Apigenin treatment also led to the induction of apoptosis, evidenced by the cleavage of poly ADP-ribose polymerase, a marker of apoptosis [213]. This cleavage was concentration-dependent, suggesting that higher doses of apigenin are more effective at inducing cell death in glioblastoma cells [213]. Furthermore, flow cytometry analysis revealed that apigenin caused cell cycle arrest at the G2/M phase [213]. This means that the cells were prevented from progressing to the next phase of division, which contributes to the inhibition of tumor growth [213]. The lactate dehydrogenase (LDH) assay indicated that apigenin increased cytotoxicity in glioblastoma cells [213]. The release of LDH, which signifies cell membrane damage, was significantly higher in cells treated with apigenin compared to untreated controls, particularly at higher concentrations [213]. Apigenin was also shown to inhibit the phosphorylation of key proteins involved in the epidermal growth factor receptor (EGFR) signaling pathway, such as AKT and mTOR [213]. This inhibition is crucial because EGFR signaling is often overactive in glioblastoma, promoting cell survival and proliferation [213]. The study also noted a reduction in the expression of the antiapoptotic protein Bcl-xL following apigenin treatment [213]. This reduction suggests that it may promote apoptosis by disrupting the balance between pro-apoptotic and anti-apoptotic signals in glioblastoma cells [213].

The study by Lotfi et al. sheds light on the potential of apigenin and its dimeric forms as therapeutic candidates for glioblastoma multiforme by targeting PIM1 kinase [214]. The research found that PIM1 kinase expression was significantly elevated in GBM tumor tissues compared to normal brain tissues, and this overexpression was associated with poorer survival rates [214]. Molecular docking studies revealed that both apigenin and its dimeric flavonoids, such as amentoflavone and hinokiflavone, could effectively bind to the ATP-binding site of PIM1 kinase [214]. Notably, the dimeric flavonoids exhibited a stronger binding affinity to PIM1 compared to apigenin itself, suggesting that the dimeric forms may be more effective in inhibiting PIM1 activity, which could enhance their therapeutic potential against GBM [214]. In the context of glioblastoma, PIM1 inhibition is expected to lead to tumor growth suppression, cell cycle arrest, and promotion of apoptosis in cancer cells [214].

Apigenin has shown promise in enhancing the effects of radiation therapy on glioma stem cells [215]. The combination of apigenin and radiation significantly reduced the viability, colony formation, and migration of both human GSCs SU3 and its radioresistant line SU3-5R, suggesting that apigenin can indeed enhance the effectiveness of radiation therapy in treating glioma stem cells [215]. Moreover, apigenin, in conjunction with radiation, led to a decrease in radiation-induced lactic acid production, which is a marker of glycolytic activity [215]. This indicates that apigenin may help in reducing the metabolic adaptation of GSCs to hypoxic conditions [215]. Apigenin treatment also resulted in decreased expression levels of key proteins associated with glycolysis and tumor progression, including HIF-1α, GLUT-1/3, NF-κB p65, and PKM2 [215]. These reductions suggest that apigenin may inhibit the glycolytic pathway by targeting these proteins, which are crucial for the survival and proliferation of GSCs under hypoxic conditions [215]. Further experiments using a hypoxia-induced model confirmed that apigenin effectively reduced the expression of HIF-1α and other glycolytic proteins in both SU3 and SU3-5R cells, reinforcing the idea that apigenin’s mechanism of increasing radiosensitivity is linked to its ability to attenuate glycolysis through HIF-1α inhibition [215].

#### 4.7.5. Acacetin

Acacetin and apigenin demonstrated selective cytotoxicity towards U87 glioblastoma cells, with relatively low IC50 values, indicating that these compounds can effectively inhibit the growth of glioblastoma cells [216]. Treatment with these flavones led to a significant G2/M phase arrest in the cell cycle, confirmed by flow cytometry, which also showed an increase in the sub G1 population, indicating apoptosis [216]. Annexin-V-FLUOS and DAPI staining further confirmed that both compounds induce apoptosis in U87 cells, suggesting they halt cell division and trigger programmed cell death [216]. The compounds were also found to induce the production of reactive oxygen species in the glioblastoma cells, linking ROS generation to their anticancer effects [216]. Acacetin upregulated p21 and downregulated Cyclin-A1, Cyclin-B1, and Cdk-1, contributing to G2/M phase arrest, while apigenin activated both the extrinsic and intrinsic pathways of apoptosis, as evidenced by the regulation of proteins such as Bax, t-Bid, caspase 8, caspase 9, caspase 3, and PARP [216]. These effects were statistically significant, highlighting the potential of acacetin and apigenin as therapeutic agents against glioblastoma [216].

#### 4.7.6. Irigenin

Irigenin exhibits significant anti-tumor activity against glioblastoma cells by inhibiting GBM cell proliferation in a dose-dependent manner and inducing cell cycle arrest at the G2/M phase [217]. Moreover, irigenin triggers apoptosis in GBM cells and inhibits their migration, which is crucial in preventing cancer metastasis [217]. Mechanistically, irigenin treatment leads to a decrease in the expression of YAP and suppresses β-catenin signaling, pathways known to be involved in cancer progression [217]. The anti-tumor effects of irigenin on GBM cells can be partially restored by overexpressing YAP, indicating YAP’s critical role in mediating irigenin’s effects [217]. In vivo studies using a GBM xenograft mouse model further confirmed that irigenin inhibits tumor growth through the inactivation of YAP [217].

#### 4.7.7. Kaempferol

Kaempferol (Kae) demonstrates significant anti-glioblastoma activity by targeting multiple mechanisms [218]. It inhibits the growth of glioblastoma cells (U87MG and U251) in a dose-dependent manner, with IC50 values of 97.2 µM for U87MG cells and 79.2 µM for U251 cells [218]. In a mouse model, kaempferol treatment (40 mg/kg) notably reduced tumor size without affecting the weight of the mice, suggesting it may be a safe anti-glioma therapy [218]. The compound increases the levels of ROS in glioblastoma cells, which is linked to the induction of cell death processes like apoptosis and pyroptosis [218]. Kaempferol treatment also leads to a decrease in mitochondrial membrane potential, promoting the release of proteins that induce cell death [218]. Additionally, it promotes autophagy in glioblastoma cells, indicated by increased expression of autophagy markers, and induces pyroptosis, evidenced by increased cleavage of gasdermin E and elevated levels of pro-inflammatory factors [218]. The autophagy induced by kaempferol may lead to pyroptosis, as inhibition of autophagy reduces the levels of pyroptosis markers, indicating a regulatory relationship between these two processes [218].

Kae promotes apoptosis in glioma cells and induces cell cycle arrest specifically in the G2/M phase [219]. Additionally, it effectively reduces the migratory and invasive capabilities of glioma cells, particularly at lower concentrations [219]. Network pharmacology and molecular docking analyses have identified EGFR and SRC as direct molecular targets of Kae in glioma therapy [219]. Kae significantly inhibits the phosphorylation of EGFR, SRC, and STAT3, suggesting that it exerts its antitumor effects by disrupting these critical signaling pathways [219]. The combination of Kae and Gef (gefitinib) results in a more pronounced inhibition of glioma cell proliferation compared to either treatment alone, and Kae further enhances the inhibition of EGFR and SRC phosphorylation when used alongside Gef [219]. These antiglioma effects of Kae have been confirmed in vivo using xenograft tumor nude mice models, demonstrating that Kae effectively inhibits tumor growth and enhances the antitumor effects of Gef by targeting the EGFR/SRC/STAT3 signaling pathway [219].

Enzymatic hydrolysis can enhance the anticancer properties of kaempferol [220]. Santos et al. successfully used hesperidinase and β-galactosidase to modify an extract from *Sophora japonica* (KPF-BBR), creating a more potent form of kaempferol (KPF-ABR) compared to the original extract [220]. Both KPF-BBR and KPF-ABR reduced the number of viable glioma cells (NG-97 and U251), with KPF-BBR having an IC50 of 800 µM for NG-97 and 1800 µM for U251, while KPF-ABR had an IC50 of 600 µM for NG-97 and 1800 µM for U251 [220]. KPF-ABR was particularly effective in triggering apoptosis, especially in the NG-97 cell line, which is less resistant to treatments [220]. Flow cytometry confirmed that both KPF compounds led to significant cell death through apoptosis [220]. Both KPF-BBR and KPF-ABR caused cell cycle arrest in the S and G2/M phases in U251 cells, effectively stopping the cancer cells from dividing and growing [220]. Additionally, both extracts significantly slowed down the migration of glioma cells, with KPF-ABR being more effective than KPF-BBR in this regard, and reduced the formation of neurospheres, suggesting they may target cancer stem-like cells [220].

#### 4.7.8. 2-(3,4-Dihydroxyphenyl)-5,7-dihydroxy-4H-chromen-4-one

The study by Rauf et al. highlights the anticancer potential of flavonoids isolated from *Pistacia chinensis* against the glioblastoma U87 cell line [221]. Compound 1, identified as 2-(3,4-dihydroxyphenyl)-5,7-dihydroxy-4H-chromen-4-one, significantly reduced cell viability in vitro, achieving up to a 70.11% reduction after 48 h at a concentration of 75 µg/mL [221]. Molecular docking studies indicated that Compound 1 has a strong binding affinity to the mTOR protein, with a binding energy of −10.391 kcal/mol, suggesting it may effectively inhibit mTOR, a key player in cancer cell growth and proliferation [221]. Density Functional Theory analysis confirmed the structural stability of Compound 1, and favorable ADMET (absorption, distribution, metabolism, excretion and toxicity) profiles suggest its potential for further development as a therapeutic candidate for glioblastoma [221].

#### 4.7.9. 5-Hydroxy-3′,4′,6,7-tetramethoxyflavone

Papapetrou et al. evaluated the cytotoxic effects of 5-hydroxy-3′,4′,6,7-tetramethoxyflavone (TMF) on glioblastoma cell lines U87MG and T98G, finding that TMF significantly reduced cell viability in both cell lines [222]. Flow cytometry analysis revealed that TMF treatment led to G0/G1 cell cycle arrest, suggesting that TMF may inhibit the progression of the cell cycle, which is crucial for cancer cell proliferation [222]. Furthermore, TMF treatment reduced the migratory capacity of glioblastoma cells in vitro [222]. A critical finding was the antagonistic effect of TMF when combined with radiotherapy [222]. While TMF showed promising antineoplastic effects, its interaction with radiotherapy was not beneficial, suggesting that TMF may interfere with the effectiveness of radiation treatment [222].

#### 4.7.10. Epigallocatechin Gallate

Epigallocatechin gallate (EGCG) shows the potential in combination therapy for glioblastoma by targeting the Warburg effect [223]. The triple-drug combination of EGCG, temozolomide, and metformin significantly reduced glucose uptake in glioblastoma cells and rat brain tissues, effectively inhibiting the Warburg effect [223]. This was evidenced by a significant reduction in the expression levels of key markers associated with the Warburg effect, such as GLUT1, GLUT4, PKM2, and LDHV [223]. This approach aligns with a broader understanding of metabolic modulation as a strategy to enhance the response to chemotherapy in glioblastoma [223]. Gliomblastoma is characterized by marked glycolytic metabolism, which is associated with increased invasion capacity and higher resistance to conventional treatment [223].

The study by Kuduvalli et al. demonstrates the therapeutic efficacy of a combination of metformin, EGCG, and temozolomide in a glioma-induced xenograft rat model [224]. This triple-drug therapy significantly inhibited tumor growth and resulted in a 50% increase in the survival rate of the rats [224]. The mechanism of action involves the inhibition of the PI3K/AKT/mTOR pathway, cell cycle arrest at the G1 phase, and the induction of caspases-dependent apoptosis [224]. The effectiveness of the triple-drug combination is attributed to differences in molecular interactions, potentially due to varying electrostatic potentials among the drugs [224].

The combination of Arsenic Trioxide, epigallocatechin-3-gallate, and resveratrol reduced cell proliferation, induced apoptosis, and decreased metabolic activity in glioblastoma cell lines (U87-MG, A-172, and 1321N1) highlight the potential of this combination [225]. The observed decrease in metabolic activity reductions of 45% in U87-MG, 27% in A-172, and 32% in 1321N1 cell lines after 72 h) and colony formation (reduced the number of colonies formed by 38% in the U87-MG cell line), along with the modulation of key genes (increase in the expression of the pro-apoptotic gene Caspase-3 and a decrease in the anti-apoptotic gene BCL-2, along with reduced levels of MMP-2, MMP-9, uPA, and uPAR) involved in apoptosis and invasion, supports the notion that this therapy can effectively target aggressive brain tumor cells [225].

Rahman et al. studied the synergistic effect of hydroxychavicol (HC) and epigallocatechin-3-gallate against glioma cell lines [226]. The combination of HC and EGCG at low doses exhibited strong synergism in SW1783 and LN18, and a moderate interaction in 1321N1 [226]. Both HC and EGCG alone inhibited the proliferation of glioma cell lines, and their combination significantly enhanced the suppression of glioma cell growth [226]. The combination treatment induced cell cycle arrest at the S phase and reduced migration and invasion in the 1321N1 and LN18 cell lines [226]. The study revealed that EGCG+HC treatment downregulated pathways related to axon guidance and metabolism, while also interfering with the endoplasmic reticulum’s unfolded protein response pathway [226].

#### 4.7.11. Epicatechin

Epicatechin exhibits a strong binding affinity to the MDM2-p53 complex, effectively inhibiting their interaction, which is crucial for glioblastoma progression [227]. The low Ki value of 82.34 nM suggests that only a small concentration of epicatechin is needed to achieve significant inhibition [227]. Molecular docking analysis revealed that epicatechin forms six hydrogen bonds with key residues involved in the MDM2-p53 interaction [227]. This mechanism is particularly relevant because the MDM2-p53 interaction is a well-known target in cancer therapy [227].

#### 4.7.12. Chrysin

Chrysin significantly inhibited the proliferation, migration, and invasion of glioblastoma cells by deactivating the Nrf2 signaling pathway and downregulating the phosphorylation of ERK1/2 [228]. Chrysin reduced the movement of Nrf2 into the nucleus, where it usually promotes cell survival and growth [228]. This reduction in Nrf2 activity was linked to decreased levels of certain proteins that help cancer cells thrive, such as hemeoxygenase-1 (HO-1) and NAD(P)H quinone oxidoreductase-1 (NQO-1) [228]. Moreover, chrysin downregulated the phosphorylation of ERK1/2, a part of the MAPK signaling pathway that can influence Nrf2 activity [228]. By inhibiting ERK1/2, chrysin further contributed to the reduction in Nrf2 levels, suggesting that the ERK/Nrf2 signaling pathway is a critical target for chrysin’s anticancer effects [228]. In vivo experiments with mice confirmed chrysin’s potential as a treatment in living organisms [228].

The study by Mahdi et al. highlights a promising approach to enhance chrysin’s therapeutic efficacy against glioblastoma cells through the formation of a multicomponent inclusion complex (CR-MC) [229]. The complexation of chrysin with hydroxyl propyl beta cyclodextrin and L-arginine significantly increased its solubility and bioavailability [229]. The dissolution study showed a substantial increase in chrysin release from the CR-MC compared to its physical mixture and pure chrysin [229]. Characterization techniques confirmed that chrysin was converted into an amorphous form, enhancing its solubility without altering its chemical structure [229]. The CR-MC demonstrated enhanced activity against the human primary glioblastoma cell line (U87-MG), reducing cell viability and inducing apoptosis [229]. This suggests that the CR-MC could be a promising formulation for improving the therapeutic efficacy of chrysin in cancer treatment [229].

#### 4.7.13. Xanthohumol

Xanthohumol demonstrates a significant anti-tumor effect on glioblastoma by directly inhibiting glycolysis [230]. It suppresses the proliferation of GBM cells and reduces colony formation [230]. Xanthohumol impairs glucose metabolism in GBM cells by inhibiting the expression of Hexokinase 2, a key enzyme in the glycolytic pathway [230]. The downregulation of c-Myc is essential for the decrease in HK2 levels induced by xanthohumol [230]. Moreover, xanthohumol destabilizes c-Myc and promotes its ubiquitination through the FBW7 pathway [230]. It attenuates Akt activity and inhibits GSK3β activation, leading to the degradation of c-Myc [230]. Overexpression of Myr-Akt1 can significantly rescue the inhibition of c-Myc and the suppression of glycolysis caused by xanthohumol, highlighting the importance of the PI3-K/Akt-GSK3β-FBW7 signaling axis in this process [230]. Animal studies demonstrate that xanthohumol substantially inhibits tumor growth in vivo [230].

Festa et al. found that xanthohumol induces apoptosis in human malignant glioblastoma cells through ROS generation and MAPK pathway activation [231]. It significantly decreased the viability of T98G human malignant GBM cells in a time- and concentration-dependent manner [231]. This apoptosis is linked to the activation of caspase-3 and caspase-9, along with cleavage of PARP, indicating that the mitochondrial pathway is engaged in the apoptosis process [231]. The treatment leads to mitochondrial depolarization and cytochrome c release, which are critical events in the intrinsic pathway of apoptosis [231]. The antiapoptotic protein Bcl-2 is downregulated, further supporting the pro-apoptotic effects of xanthohumol [231]. Moreover, it induces the production of intracellular ROS, essential for activating the mitochondrial pathway and subsequent apoptosis [231]. The antioxidant N-acetyl-L-cysteine can reduce ROS levels, demonstrating the importance of oxidative stress in this process [231]. Oxidative stress from xanthohumol treatment is associated with the activation of MAPK pathways, specifically through the phosphorylation of ERK1/2 and p38 MAPK [231]. The use of specific inhibitors for ERK1/2 and p38 showed that the MAPK pathway played a significant role in mediating cell death induced by xanthohumol, with the effects being reduced by these inhibitors [231].

#### 4.7.14. Hispidulin

Hispidulin enhances the effectiveness of temozolomide against glioma cells by inhibiting autophagy and promoting apoptosis [232]. When combined with TMZ, hispidulin significantly reduces the viability of U87MG glioma cells, suggesting that it can make cancer cells more sensitive to TMZ, leading to increased cell death [232]. Hispidulin increases the levels of proteins associated with apoptosis (Bax, cleaved-caspase-3, and cleaved-caspase-9) while decreasing the levels of the anti-apoptotic protein Bcl-2 [232]. in addition, it inhibits the autophagy induced by TMZ, preventing cancer cells from using this survival mechanism [232]. Fewer acidic vesicular organelles, which are markers of autophagy, are observed in cells treated with both hispidulin and TMZ [232]. This combination could be a promising strategy for treating glioma patients who show resistance to TMZ [232].

#### 4.7.15. Silibinin

Silibinin significantly inhibits the proliferation of glioblastoma cells [233]. Liu et al. demonstrated it through cell counting kit-8 assays and EdU assays, which measure cell viability and proliferation rates, respectively [233]. Silibinin promotes apoptosis in GBM cells, as assessed using flow cytometry [233]. It reduces the invasive capabilities of GBM cells and diminishes their stemness properties, evaluated using transwell assays and sphere formation assays [233]. It also affects glutamine metabolism in GBM cells by inhibiting glutamine consumption, glutamate production, and α-ketoglutarate production [233]. Moreover, the study identifies that SLC1A5, a solute carrier protein, is upregulated in GBM and its expression is decreased by Silibinin [233]. The transcription factor YY1 binds to the promoter region of SLC1A5, inducing its expression [233]. Silibinin’s anti-tumor effects are reversed by SLC1A5 overexpression, indicating a critical role of this pathway in GBM progression [233]. Mice xenograft models demonstrate that Silibinin reduces tumor growth in vivo, further supporting its potential as a therapeutic agent against GBM [233].

Silibinin significantly inhibits the metabolic activity of human glioblastoma cells in a concentration-dependent manner, with over 80% of cells losing viability after 24 h of treatment with 100 µM silibinin [234]. Silibinin induces apoptosis in glioblastoma cells, confirmed by flow cytometry, with the mechanism involving the cleavage of caspase-3 and PARP [234]. Interestingly, silibinin also induces autophagy, and when autophagy is inhibited using chloroquine, the apoptosis induced by silibinin is significantly enhanced, suggesting a protective role of autophagy against cell death in this context [234]. It inhibits the phosphorylation of mTOR and downregulates YAP, affecting multiple signaling pathways that contribute to glioblastoma cell survival [234]. Overall, silibinin likely inhibits glioma cell proliferation and induces apoptosis through inactivation of PI3K and FoxM1, leading to activation of the mitochondrial apoptotic pathway [234].

### 4.8. Resveratrol

Resveratrol, a naturally occurring polyphenolic compound found in plants like grapes, berries, and nuts, has been widely studied for its broad spectrum of beneficial biological effects [235,236,237]. These encompass its potent antioxidant properties, anti-inflammatory activities, anti-aging capabilities, cardioprotective functions, and neuroprotective potential [235,236,238]. Furthermore, resveratrol has been shown to exert effects on metabolic processes and demonstrate the ability to effectively traverse the blood–brain barrier [235,236].

Resveratrol (RSV) demonstrates potential as an effective therapeutic agent against glioblastoma [239]. RSV significantly reduced the viability of GBM cells, particularly in the LN-229 and U87-MG cell lines, in a dose- and time-dependent manner [239]. Resveratrol treatment promoted apoptosis in GBM cells by upregulating pro-apoptotic proteins like Bax and downregulating anti-apoptotic proteins like Bcl-2 [239]. Furthermore, it inhibited the migration and invasion abilities of GBM cells and improved the inflammatory microenvironment by inhibiting the NLRP3 inflammasome and the JAK2/STAT3 signaling pathway, which is known to play a role in promoting inflammation and tumor progression [239].

Jin et al. found that resveratrol inhibits cell viability and induces apoptosis in glioblastoma A172 cells [240]. RSV treatment leads to mitochondrial dysfunction and activates caspase-9 activity, while also increasing the expression of Pak2 [240]. Inhibiting Pak2 further augments the proapoptotic effect of resveratrol, enhancing its anticancer properties [240]. The enhanced effect of Pak2 inhibition in combination with resveratrol involves several mechanisms, including overproduction of reactive oxygen species, activation of the mitochondria-JNK pathway, and suppression of the AMPK-YAP axis [240]. The AMPK-YAP pathway plays a necessary role in regulating the cancer-suppressive actions of resveratrol and Pak2 inhibition, as overexpression of YAP can abolish their anticancer effects [240].

Resveratrol demonstrates antitumoral effects also on C6 glioma cells by interacting with adenosine signaling and metabolism [241]. Resveratrol treatment significantly diminished C6 glioma cell viability and proliferation in a time- and concentration-dependent manner, with 100 µM RSV for 24 h reducing cell viability by approximately 50% [241]. The population doubling time also increased significantly with 100 µM RSV treatment [241]. Furthermore, RSV exposure led to an accumulation of C6 glioma cells in the G1 phase, indicating an antiproliferative effect through cell cycle arrest [241]. In terms of adenosine metabolism, RSV treatment significantly reduced the activity of 5′-nucleotidase and adenosine deaminase, leading to increased extracellular adenosine levels and reduced intracellular inosine levels [241]. The blockade of A1 and A3 receptors during RSV treatment significantly reduced RSV’s effect on cell viability and cell number, suggesting their involvement in RSV’s antiproliferative action [241]. Despite the increase in extracellular adenosine, adenosine alone was unable to mimic the full antitumoral effect of RSV on cell viability, suggesting that RSV’s action is not solely dependent on increased extracellular adenosine [241].

Jia et al. highlight the variable effects of resveratrol on glioblastoma cells, specifically comparing U251 and LN428 cell lines [242]. They found that resveratrol increased oxidative stress in glioblastoma cells, particularly in U251 cells, leading to apoptosis, but LN428 cells did not respond similarly [242]. In U251 cells treated with resveratrol, there was a notable increase in reactive oxygen species levels over time, indicating heightened oxidative stress [242]. This was accompanied by mitochondrial damage, characterized by the formation of spheroid mitochondria [242]. Additionally, the expression of antioxidant enzymes, specifically superoxide dismutase-2 and catalase, decreased in U251 cells after resveratrol treatment, suggesting that the antioxidant defense system in these cells was overwhelmed, contributing to increased oxidative damage [242]. The activation of caspases, proteins that play essential roles in programmed cell death (apoptosis), was significantly higher in U251 cells treated with resveratrol, indicating that resveratrol induced apoptosis through a mitochondrial pathway [242]. In conclusion, the study suggests that resveratrol can increase oxidative stress in glioblastoma cells, particularly in U251 cells, leading to apoptosis, and that the effectiveness of resveratrol as a treatment may depend on the specific characteristics of the cancer cells, highlighting the importance of understanding individual cell responses for personalized therapy [242].

Liu et al. found that a low-dose combination of 25 µM resveratrol (Res) and 250 µM TMZ effectively suppressed the growth of RG-2 cells, a rat glioblastoma cell line [243]. This combination also showed similar suppressive effects to higher individual doses of Res (100 µM) and TMZ (500 µM) [243]. In overcoming resistance, some GBM cell lines (LN-18 and LN-428) were less sensitive to single treatments of resveratrol or TMZ; however, their growth was significantly suppressed when treated with higher-dose combinations, such as 75 µM Res and 750 µM TMZ [243]. That indicates that Res/TMZ combinations can overcome primary and secondary drug resistance in GBM cells [243]. The combination of Res/TMZ leads to a high percentage of DNA damage and apoptosis in RG-2 cells and also induces apoptosis in LN-18 and LN-428 cells [243]. It also causes cell cycle arrest, specifically increasing the proportion of RG-2 cells in the S phase and LN-18 cells in the G0/G1 phase [243]. In terms of underlying mechanisms, resveratrol has a regulatory effect on the expression of O6-methylguanine-DNA methyltransferase, a key enzyme associated with TMZ resistance [243]. Resveratrol can significantly downregulate MGMT overexpression caused by TMZ [243]. For example, treatment with 25 µM Res reduced MGMT expression in RG-2 cells, and the combination of 25 µM Res/250 µM TMZ further decreased it compared to TMZ alone [243]. Additionally, the STAT3 (signal transducer and activator of transcription 3) signaling pathway, which is often highly active in glioma cells and contributes to drug resistance, was remarkably inhibited in Res/TMZ-treated GBM cells [243]. This inhibition included a reduction in STAT3 expression and its phosphorylation [243]. The levels of survivin and Bcl-2, which are active in cell proliferation and maintenance of gliomas and are target genes of the STAT3 pathway, were decreased in Res/TMZ-treated cells [243]. Frequent upregulation of MGMT and activation of STAT3 are unfavorable factors in GBM treatment, and studies suggest they may be potential targets for Res/TMZ therapy [243]. The combination treatment of resveratrol and temozolomide demonstrates synergistic effects by inhibiting MGMT expression and downregulating the STAT3/Bcl-2/survivin signaling pathway, which leads to increased apoptosis and cell cycle arrest in glioma cells [243]. Given the challenges of GBM treatment, including high recurrence rates and drug resistance, the synergistic effects of Res/TMZ offer a promising strategy to improve sensitivity and potentially reverse TMZ resistance [243]. The ability to achieve anticancer effects with lower doses of TMZ when combined with resveratrol could also improve patients’ quality of life by reducing side effects [243].

The growth inhibition rate for A172 cells treated with both Res and TMZ was 70.2%, higher than with either drug alone [244]. Similarly, LN428 cells showed improved sensitivity to TMZ when treated with Res, suggesting this combination could benefit patients resistant to TMZ alone [244]. Resveratrol likely works by downregulating STAT3, a protein often activated in GBM and associated with poor prognosis [244]. The combination of Res and TMZ led to a more significant reduction in STAT3 levels compared to either treatment alone [244]. Furthermore, the combination treatment effectively reduced MGMT (O6-methylguanine-DNA methyltransferase) levels in LN428 cells, potentially explaining the reversal of resistance observed in these cells, whereas TMZ alone had little effect on MGMT levels [244]. The increased rates of apoptosis in both A172 and LN428 cells with the combination therapy, as compared to single treatments, suggest that Res not only enhances TMZ’s effectiveness but also promotes cancer cell death [244].

Resveratrol significantly enhances the effectiveness of radiotherapy in treating glioblastoma by promoting apoptosis, reducing tumor growth, and improving immune responses [245]. Combining radiation and resveratrol led to increased body weight and improved survival rates in rats, with no deaths in the cotreatment group [245]. The combination also significantly reduced tumor volume more effectively than either treatment alone, suggesting that resveratrol acts as a radiosensitizer [245]. Histological analysis showed less pathological damage in brain tissue in the cotreatment group, evidenced by reduced tumor cell infiltration and lower expression levels of Ki-67 and PD-L1 [245]. Moreover, the cotreatment led to a higher rate of apoptosis in tumor cells and caused cell cycle arrest, with resveratrol enhancing antitumor immunity by increasing the percentages of CD3 + CD8 + T cells and IFN-γ + CD8 + T cells while decreasing regulatory T cells [245]. These findings suggest that resveratrol may help the immune system better target and destroy tumor cells [245].

The study by Yi et al. investigates the effectiveness of intratumor injection of interferon-elastin-like polypeptide (IFN-ELP(V)) combined with resveratrol in treating glioblastoma, particularly in patients who cannot undergo surgical resection [246]. The combination of IFN-ELP(V) and Res significantly delays the growth of GBM in mice models and increases median survival rates in an orthotopic GBM model [246]. IFN-ELP(V) is a biodegradable, thermosensitive fusion protein that promotes antitumor immunity, and resveratrol is known for its antitumor effects [246]. The study indicates that these two agents work synergistically to enhance therapeutic outcomes against GBM [246].

In the treatment of glioblastoma using intranasal delivery for brain targeting, resveratrol’s synergistic activity with temozolomide has led to its co-loading into nanostructured lipid carriers to enhance their combined anticancer effects [247]. An optimized lactoferrin-conjugated NLC (LTR-NLC) formulation achieved a high entrapment efficiency of 87.59% for resveratrol, indicating successful incorporation into the nanoparticles [247]. Ex vivo permeation studies demonstrated a substantial increase in resveratrol’s permeation when delivered via LTR-NLC, with a nearly 3-fold increase in penetration compared to a drug suspension [247]. This LTR-NLC formulation also led to a significantly better IC50 value in treated groups, suggesting improved anticancer activity [247].

### 4.9. Rosmarinic Acid

Rosmarinic acid (RA), a caffeic acid ester, is a naturally occurring phenolic compound present in several members of the Lamiaceae family, such as Rosmarinus officinalis (rosemary), where it was originally identified [248]. RA may influence oxidative stress levels within the glioblastoma environment, potentially affecting tumor progression and survival outcomes [249]. Studies measuring the activities of antioxidant enzymes, specifically superoxide dismutase (SOD) and catalase (CAT), in tumor tissue after RA administration have shown a significant reduction in their activity, which was associated with a decrease in tumor volume [249]. Histological analysis also revealed less invasion of tumor cells into the normal brain tissue in rats treated with RA, which may relate to the modulation of oxidative stress [249]. These results suggest that RA has a potential role in managing oxidative stress in glioblastoma, which could be a critical factor in its anticancer properties [249]. By affecting oxidative stress pathways, RA may help in controlling cell proliferation and promoting apoptosis in tumor cells, thereby enhancing survival rates in animal models [249].

### 4.10. Sinularin

Sinularin is a natural product extracted from soft coral and is shown to exhibit antitumor effects against multiple human cancers [250]. Hsu et al. found that sinularin treatment in glioblastoma cells leads to the generation of reactive oxygen species, which is critical for subsequent cell death [251]. The oxidative stress caused by ROS generation is linked to mitochondria-mediated apoptosis, evidenced by the upregulation of cleaved forms of pro-apoptotic proteins such as caspase 9, caspase 3, and PARP [251]. Additionally, sinularin treatment results in a downregulation of antioxidant enzymes, specifically SOD1/2 and thioredoxin, which compromises the cells’ ability to combat oxidative stress, further contributing to the apoptotic process [251]. Sinularin treatment also results in significant reductions in mitochondrial respiration capacities, a direct consequence of the oxidative stress induced by sinularin [251]. These findings indicate that sinularin not only induces oxidative stress but also leads to mitochondrial dysfunction and apoptosis in glioblastoma cells, highlighting its potential as a therapeutic agent that targets oxidative stress pathways in cancer treatment [251].

### 4.11. Vitamin A

Vitamin A is a crucial fat-soluble vitamin that plays a significant role in various physiological functions, including vision, immune response, reproduction, and skin health [252,253]. It exists in two primary forms: preformed vitamin A (retinoids) found in animal products, and provitamin A (carotenoids) found in plant-based foods [252,253]. Vitamin A derivatives demonstrate significant anticancer potential in glioblastoma by promoting cell differentiation, inhibiting stemness, inducing apoptosis, and suppressing invasion and migration [254].

Vitamin A, primarily in the form of retinoids, has shown promising effects on glioblastoma cells, influencing various cellular processes such as differentiation, proliferation, and apoptosis [254]. Retinoids activate cell differentiation and suppress biomarkers responsible for stemness in human GBM cells, potentially reducing drug resistance [254]. Treatment with all-trans retinoic acid (ATRA) inhibits the expression of stemness markers like CD133 and Sox2 in stem-like cells derived from GBM, promoting cell differentiation, and reverses the suppression caused by NSPc1 on RDH16, allowing differentiation to occur [254]. ATRA induces cell differentiation through the activation of the extracellular signal-regulated kinase 1/2 (ERK1/2) pathway and leads to the overexpression of glial fibrillary acidic protein in GBM cells [254]. ATRA can also trigger apoptotic pathways, particularly in tumors that express PTEN, by modulating the expression of several biomarkers associated with apoptosis, including an increased Bax:Bcl-2 ratio, cytosolic release of cytochrome C, and activation of Caspase-3 [254]. Additionally, ATRA causes cell cycle arrest at the G1/S phase by upregulating cyclin-dependent protein kinase inhibitor 1B (CDKN1B) ‘p27kip1’ and subsequently suppressing CDK2 in U87MG cells [254]. Furthermore, it inhibits cell invasion and migration in GBM cells by suppressing matrix metalloproteinase-2 and MMP-9 [254]. However, the accumulation of the transporter protein CRABP2 in the cytoplasm of GBM cells can block the action of retinoic acid, leading to a failure in retinoid transport to the nucleus and hindering its growth and proliferation-ceasing effects [254]. Changes in apoptotic markers are not consistently observed in all GBM cell lines, particularly in U87G cells which do not express PTEN [254]. CRABP2 accumulation can limit retinoids efficacy, what highlights the need for further research into their molecular mechanisms and potential for combination therapies [254].

### 4.12. Vitamin E

Vitamin E is a collective term for a group of fat-soluble compounds, primarily two main forms: tocopherols and tocotrienols, which are essential for various biological functions in mammals [254,255,256,257,258]. Each form has four natural isoforms: alpha (α), beta (β), delta (δ), and gamma (γ), resulting in eight naturally occurring derivatives [254]. The dietary sources of vitamin E include vegetable oils, seeds, and cereal grains, with α-tocopherol being predominantly found in wheat germ, sunflower, and safflower oils [254,257,258,259]. The most biologically active form of vitamin E is α-tocopherol, which plays a crucial role in protecting cells from oxidative damage by scavenging free radicals [255,257,258]. This antioxidant property is fundamental in preventing lipid peroxidation in cell membranes and lipoproteins, thereby contributing to the prevention of chronic diseases such as cardiovascular diseases, cancer, and neurodegenerative disorders [255,256,257,258]. In glioblastoma, vitamin E has been investigated for its potential role primarily due to its antioxidant properties [254].

Vitamin E derivatives demonstrate anticancer effects against glioblastoma cells through multiple mechanisms [254]. Alpha- and gamma-tocopherols inhibit U87MG GBM cell proliferation, while tocotrienol isoforms (alpha-, gamma-, and delta-T3) show even more potent growth inhibition in a time- and concentration-dependent manner, with delta-T3 exhibiting the highest potency [254]. These compounds induce cell cycle arrest through different pathways: alpha-tocopherol arrests cells by inhibiting protein kinase C (PKC) and reducing cyclin D1 and cyclin E levels, while tocotrienols arrest GBM cells at the pre-G1 phase [254]. Regarding apoptosis, tocotrienols, particularly gamma-T3, effectively activate both intrinsic and extrinsic apoptotic pathways by upregulating pro-apoptotic proteins like Bax and TRAIL, inducing Caspase-3 and Caspase-8 activity, and increasing levels of Bax, Bid, and cytosolic cytochrome c [254]. However, tocopherols show inconsistent apoptotic effects when used alone, though alpha-tocopherol combined with low-dose methotrexate significantly increases cell death and apoptotic biomarkers [254]. Additionally, alpha- and gamma-tocopherols reduce cell invasion and migration by decreasing cell adhesion to fibronectin and increasing integrin expression, while alpha-tocopheryl succinate demonstrates chemosensitization by reducing multidrug resistance protein 1 expression and intracellular glutathione levels [254]. Despite these promising effects, the anticancer properties of tocopherols can be inconsistent, as exemplified by gamma-tocopherol’s ability to simultaneously inhibit proliferation while increasing cell migration, highlighting the need for further research to optimize their therapeutic application in GBM treatment [254].

In the research by Hsu et al., vitamin E’s role in ameliorating chlorpyrifos-induced cytotoxicity has been explored in the context of human glioblastoma cells [260]. The study indicates that vitamin E can reverse the cytotoxicity and oxidative stress caused by chlorpyrifos treatment in DBTRG-05MG cells [260]. The protective mechanism of this vitamin involves the modulation of apoptosis-related proteins, such as Bax, Bcl-2, and caspases, which are crucial in the apoptotic pathways activated by oxidative stress [260].

Lin et al. investigated the cytotoxicity induced by bioallethrin, a pyrethroid insecticide, in human glioblastoma cells [261]. The research found that bioallethrin increased the production of reactive oxygen species, leading to cytotoxic effects [261]. This increase in ROS was linked to a decrease in glutathione levels, an important antioxidant [261]. The study also demonstrated that pretreatment with Vitamin E partially reversed the cytotoxic effects caused by bioallethrin, suggesting that Vitamin E can mitigate the oxidative stress and subsequent apoptosis triggered by bioallethrin in the glioblastoma cells [261]. These findings support the conclusion that Vitamin E has a protective role against oxidative stress induced by bioallethrin [261]. By reducing ROS levels and modulating the expression of apoptosis-related proteins, Vitamin E may help in lessening the brain damage associated with exposure to bioallethrin [261].

While in vitro studies show promising anticancer effects of vitamin E derivatives against glioblastoma cells, epidemiological studies present a more complex picture [262]. A meta-analysis by Ni et al. found no significant correlation between Vitamin E consumption and glioma risk, suggesting that the protective effects observed in cell models may not directly translate to population-level outcomes [262].

### 4.13. Vitamin C

Vitamin C (ascorbic acid)—a water-soluble nutrient-serves as a vital compound with multiple functions in human health [263,264,265]. Its essential biochemical roles include facilitating collagen synthesis, enhancing iron absorption, and providing antioxidant protection [264,265]. Due to humans lacking the enzyme L-gulono-gamma-lactone oxidase, unlike many other animals, the body cannot produce vitamin C internally, necessitating adequate dietary consumption [263,265]. Rich dietary sources of this nutrient include fruits and vegetables, particularly citrus fruits, berries, and cruciferous vegetables [265]. Beyond its fundamental nutritional importance, vitamin C contributes to skin health maintenance, supports immune system function, and may influence DNA and histone structural modifications [263,265]. Research indicates that vitamin C can influence glioblastoma progression through various mechanisms, including its antioxidant properties, effects on tumor cell proliferation, and modulation of epigenetic and hypoxic pathways [262,266,267].

Piotrowsky et al. studied the effects of high-dose ascorbate on human glioblastoma cell lines, revealing significant findings regarding its cytotoxicity, antiproliferative efficacy, and mechanism of action [268]. High-dose ascorbate demonstrated high cytotoxicity and antiproliferative efficacy in human glioblastoma cells, while dehydroascorbic acid showed much weaker effects [268]. Ascorbate-induced cell death was found to be independent of apoptosis, with the cell death mechanism caused by high-dose ascorbate in glioblastoma cells showing evidence of ferroptosis and confirmed to be caspase-3 independent [268]. Moreover, pre-treatment of tumor cells with ferric iron significantly increased both the reduction in cell viability and the ascorbate-induced generation of intracellular reactive oxygen species [268]. This highlights that high-dose ascorbate is highly effective against human glioblastoma cells, inducing cell death primarily through a ferroptosis-like mechanism, independent of apoptosis, with its efficacy significantly enhanced by pre-treatment with ferric iron, which boosts intracellular ROS formation and cytotoxicity [268].

Jara et al. investigated the impact of vitamin C deficiency on glioblastoma progression using a guinea pig model [267]. The study found that the anti-tumor effect of vitamin C deficiency was dependent on the specific tumor cell line used for GBM induction [267]. In U87-MG tumors, vitamin C deficiency led to a reduction in glomeruloid vasculature and a decrease in microglia/macrophage infiltration [267]. For tumors derived from HSVT-C3 cells, vitamin C deficiency resulted in a reduction in tumor size, proliferation, glomeruloid vasculature, microglia/macrophage infiltration, and invasion [267]. HSVT-C3 cells, characterized by stem cell features and isolated from a human subventricular GBM, showed greater sensitivity to the vitamin C-deficient condition [267]. Despite the varying sensitivity, vitamin C deficiency consistently displayed an antitumor effect in both GBM models analyzed [267]. Overall, the study indicates that vitamin C deficiency can have an antitumor effect on glioblastoma, reducing tumor growth, proliferation, vasculature, and immune cell infiltration, with a more pronounced effect observed in tumors originating from stem cell-like HSVT-C3 cells [267].

The study by Burgess et al. investigated the role of vitamin C (ascorbate) in regulating the hypoxic pathway in glioblastoma cells, focusing on the sodium-dependent vitamin C transporter-2 [269]. The researchers found that ascorbate uptake varied among different glioblastoma cell lines (U87MG, U251MG, T98G), with uptake rates ranging from 1.7 to 11.0 nmol/10^6^ cells [269]. Glioblastoma cells with SVCT2 knocked out accumulated significantly less intracellular ascorbate (90–95% less) compared to their parental counterparts, indicating that SVCT2 is crucial for ascorbate accumulation within these cells [269]. Vitamin C was also found to reduce the induction of the hypoxic pathway stimulated by cobalt chloride, desferrioxamine, or 5% oxygen [269]. However, in SVCT2-knockout cells, the suppressive effect of ascorbate on the hypoxic pathway was significantly limited, highlighting the necessity of intracellular ascorbate, transported via SVCT2, for its regulatory function [269]. In conclusion, the study verifies that intracellular ascorbate is essential for suppressing the hypoxic pathway in glioblastoma cells [269]. The findings suggest that targeting SVCT2 or increasing intra-tumoral ascorbate levels could be of clinical interest, especially given that patient survival in glioblastoma is linked to an activated hypoxic pathway [269].

Low-dose ascorbate, at sub-pro-oxidant concentrations, enhances Ten-Eleven Translocation (TET) enzymatic activity, leading to an increase in global 5-hydroxymethylcytosine (5hmC) levels and a decrease in 5-methylcytosine (5mC) in glioblastoma stem cells [270]. These changes in DNA methylation correlate with a decrease in the self-renewal capacity of GSCs [270]. Moreover, ascorbate treatment increases tumor cell death when combined with temozolomide and ionizing radiation, indicating that it sensitizes GSCs to conventional radio- and chemotherapeutics [270]. Malloy et al. identified a novel mechanism where ascorbate increases the histone mark H3K36me3 by upregulating the GSC expression of the histone methyltransferase NSD1, which is achieved through demethylation of the NSD1 gene promoter region [270]. This suggests that the enhanced susceptibility of ascorbate-treated tumor cells to DNA damage therapies results from increased H3K36me3-induced euchromatin states, which are thought to increase the genomic susceptibility to DNA damage [270].

Ascorbic acid, when combined with menadione (AA + MD), demonstrates significant anti-glioblastoma activity, primarily through the induction of oxidative stress [271]. AA + MD treatment induces severe oxidative stress within U251 human glioblastoma cells, leading to an early increase in Akt phosphorylation, followed by its strong inhibition, and persistent activation of c-Jun N-terminal kinase [271]. Ultimately, the AA + MD combination leads to U251 cell death [271]. Interestingly, small molecule Akt kinase inhibitor 10-DEBC enhances the toxicity induced by AA+MD, while pharmacological and genetic activation of Akt decreases AA + MD-induced toxicity [271]. The potentiation of U251 cell death by 10-DEBC correlates with an increase in the combination-induced autophagic flux, which is abolished by genetic autophagy silencing [271]. Additionally, the pharmacological JNK inhibitor SP600125 also augments the combination’s toxicity towards U251 cells, linked to increased ROS accumulation [271]. Overall, small Akt and JNK kinase inhibitors significantly enhance the anti-GBM effects of the menadione and ascorbic acid combination, achieved through the potentiation of autophagy and the amplification of deleterious ROS levels [271].

## 5. Challenges and Limitations

Despite their promising therapeutic effects, natural antioxidants face several significant challenges and limitations in glioblastoma therapy. Poor bioavailability is a significant hurdle, as natural antioxidants like polyphenols and flavonoids are often rapidly metabolized and degraded in the body, limiting their ability to reach therapeutic concentrations in the brain where GBM occurs [272,273,274]. The blood–brain barrier presents another obstacle, restricting the entry of many compounds into the brain, including natural antioxidants such as curcumin and resveratrol, which limits their efficacy in GBM therapy [272,275].

Furthermore, the dual role of these compounds as both antioxidants and pro-oxidants introduces complexity [112,130]. While their antioxidant effects can protect normal cells from oxidative damage, their pro-oxidant effects can selectively kill cancer cells by inducing oxidative stress [112,130]. This dual functionality can lead to conflicting outcomes in different therapeutic contexts, necessitating careful optimization of dosing and treatment regimens to maximize therapeutic benefits while minimizing potential adverse effects.

## 6. Future Directions–Combination Therapies and Novel Delivery Systems

### 6.1. Oxidative Stress

The therapeutic landscape for glioblastoma is evolving rapidly, with emerging strategies increasingly focusing on combination therapies that exploit oxidative stress mechanisms alongside novel delivery systems designed to enhance specificity, bioavailability, and blood–brain barrier (BBB) permeability [276]. Among these, redox-modulating nanotechnology combined with ferroptosis induction has garnered substantial attention [277,278]. Ferroptosis, a regulated form of cell death characterized by iron-dependent lipid peroxidation, represents a promising strategy to overcome GBM resistance to apoptosis [277,278,279]. However, the efficacy of ferroptosis-based therapies may vary significantly among GBM subtypes, with recent evidence indicating that the mesenchymal subtype exhibits enhanced antioxidant defense mechanisms that reduce susceptibility to ferroptosis-induced cell death [280]. This subtype-specific resistance highlights the importance of tailoring ferroptosis-targeting strategies according to GBM molecular classifications [280]. Several studies have developed iron oxide nanoparticle (IONP)-based systems that co-deliver chemotherapeutics and gene-silencing agents [281,282,283]. For instance, Zhang et al. designed folic acid-conjugated IONPs loaded with cisplatin and siRNA targeting GPX4 (FA/Pt-si-GPX4@IONPs), which significantly suppressed tumor growth in both U87MG and patient-derived xenograft models via synergistic ROS accumulation and ferroptosis induction, with negligible systemic toxicity [284]. Similarly, IONPs loaded with paclitaxel (IONPs@PTX) demonstrated enhanced autophagy-dependent ferroptosis and chemosensitivity in glioma cells [285]. These approaches may be particularly effective when combined with strategies to overcome the enhanced antioxidant defenses observed in mesenchymal GBM subtypes [280].

Other formulations such as cRGD/Pt + DOX@GFNPs (RPDGs) integrate glutathione (GSH) depletion, photothermal therapy (PTT), and chemotherapeutics to simultaneously trigger ferroptosis and apoptosis, demonstrating high tumor selectivity and effective imaging via MRI [286,287]. They target the intrinsic oxidative imbalance of GBM cells, which rely on elevated antioxidant defenses for survival [277]. Disruption of this redox homeostasis through GSH depletion or GPX4 inhibition renders them vulnerable to ROS-induced damage, thus offering a mechanistically rational approach for therapy [277,284].

Beyond ferroptosis-inducing constructs, biomimetic catalytic nanoplatforms have emerged that simulate enzyme activity to enhance oxidative stress and promote immunogenic cell death [288,289]. For example, a lactoferrin-modified PCN-224@Au/CeO_2_ nanoplatform mimics glucose oxidase, peroxidase, and catalase activity to supply O_2_ and catalyze ROS generation under hypoxia, thereby augmenting sonodynamic therapy (SDT) and inducing iron-independent ferroptosis [290]. Co-administration with anti-PD-L1 monoclonal antibodies enhanced CD8^+^ T-cell responses and prolonged survival in orthotopic GBM models, illustrating the potential of combining oxidative strategies with immune checkpoint inhibition [290].

Nanomotors represent a novel delivery system designed for active transport toward oxidative microenvironments [291,292]. These autonomous nanosystems can be engineered to produce hydroxyl radicals and deliver redox-active compounds such as TLND, while simultaneously releasing nitric oxide (NO), thereby integrating chemodynamic, chemotherapy, and immunotherapy in a single platform [291]. In parallel, focused ultrasound (FUS) in conjunction with microbubble-mediated BBB disruption has shown promise in enhancing the intracranial delivery of nanoparticles containing cisplatin, paclitaxel, or bevacizumab, offering a non-invasive method to improve drug penetration into gliomas [293,294]. When combined with redox-reactive nanocarriers such as MnO_2_-coated mesoporous silica or IONPs, FUS may further amplify tumor-specific oxidative damage [293,295,296].

Hydrogel-based local delivery systems also provide a means of sustained intratumoral release [297]. Thermosensitive hydrogels loaded with polymeric nanoparticles have shown prolonged retention and enhanced uptake by tumor and peritumoral tissues in orthotopic models [297,298]. When such hydrogels are embedded with redox-sensitive or GSH-responsive nanocarriers, they enable controlled release of ROS-generating agents under tumor-specific conditions, potentially enabling combination chemo-, radio-, or photodynamic therapies with minimal off-target toxicity [298].

Exosomes and extracellular vesicle (EV)-based carriers provide endogenous biocompatibility, immune evasion, and intrinsic BBB penetration [299,300]. These vesicles can be engineered to carry siRNA, drugs, or redox-regulating proteins and target tumor cells through natural ligand–receptor interactions [299,300]. EV-based delivery has been shown to reduce macrophage clearance and improve drug accumulation in GBM tissue [300]. Ferritin nanocages, another natural protein-based carrier, exploit transferrin receptor (TfR1) expression for GBM targeting [301]. Ferritin-encapsulated paclitaxel (HFn-PTX) demonstrated improved intracranial accumulation and cytotoxicity relative to free drug in preclinical models [302].

Importantly, redox-responsive nanocarriers offer tumor-triggered drug release and simultaneous GSH depletion or ROS generation [303,304]. MnO_2_ nanosheets react with intracellular GSH, triggering payload release while promoting oxidative stress [297,305]. In combination with chlorin e6 (Ce6), these systems have been employed in photodynamic therapy (PDT) to generate singlet oxygen, synergizing with ferroptosis to induce robust cell death in GBM [297,305]. Such dual-triggered platforms enable highly selective and potent therapeutic effects while minimizing systemic toxicity.

Incorporating sonodynamic, photothermal, and photodynamic therapies with redox modulation offers an additional layer of therapeutic synergy [306,307,308]. Oral administration of Ce6–gold nanoparticle conjugates functionalized with lactoferrin (Ce6-AuNP-Lf) demonstrated enhanced BBB crossing, increased ROS production, and significant tumor suppression under light activation in orthotopic GBM models [306,309]. Cascade nanozymes that generate O_2_ and ROS under SDT (sonodynamic therapy), when combined with immune checkpoint blockade, have been shown to trigger immunogenic cell death and adaptive T-cell responses, suggesting the value of integrating ROS-based therapy with immunotherapy [310,311,312].

Future directions should emphasize rational design of dual-targeting nanocarriers, employing ligands for both BBB penetration (e.g., angiopep-2, transferrin, lactoferrin) and tumor specificity (e.g., RGD peptides, EGFR, folate). Additionally, biodegradable, self-destructing systems responsive to pH, enzymes, or oxidative cues can minimize long-term nanoparticle accumulation and systemic exposure [57]. The integration of real-time imaging capabilities into therapeutic nanoplatforms will also be essential for monitoring distribution, activation, and response.

Computational modeling and artificial intelligence are poised to accelerate the development of personalized nanomedicine by optimizing carrier design, drug release kinetics, and treatment regimens. To facilitate clinical translation, future studies must prioritize the use of patient-derived xenograft (PDX) models, thorough pharmacokinetic and toxicity assessments, and standardized in vivo imaging techniques.

Combining redox-modulating strategies with nanotechnology-based delivery systems provides a highly promising avenue to enhance the efficacy and precision of GBM treatment. By leveraging the oxidative vulnerabilities of glioma cells, these platforms offer the potential to overcome resistance, penetrate the BBB, and elicit durable therapeutic responses. Continued interdisciplinary collaboration between material science, oncology, and immunology will be critical to translating these advances into clinical impact.

### 6.2. Antioxidants

To address the challenges associated with antioxidant therapy in glioblastoma, innovative strategies, enhancing the delivery and efficacy of natural antioxidants, are necessary. One approach involves developing chemical formulations such as nanoparticles, liposomes, and prodrugs to improve the bioavailability and BBB permeability of natural antioxidants [272,275]. For instance, curcumin-loaded nanoparticles have shown promise in enhancing curcumin delivery to the brain, thereby improving its therapeutic efficacy in GBM models [272]. Combination therapies that integrate natural antioxidants with conventional chemotherapeutic agents can also enhance the efficacy of glioblastoma treatment by overcoming resistance mechanisms and optimizing treatment outcomes [272,274].

Heterogeneity of glioblastoma, the complexity of redox signaling, and the potential for antioxidants to protect cancer cells under certain conditions must be carefully considered when designing therapeutic strategies. GBM’s heterogeneity poses a significant challenge for antioxidant-based therapies [125]. Therefore, stratifying patients based on their antioxidant profiles may help identify those most likely to benefit from specific treatments, which aligns with the principles of personalized medicine [125].

Rigorous clinical trials are essential to validate the efficacy and safety of natural antioxidants in humans [130,272,274,275]. They should focus on optimizing dosing regimens, evaluating the pharmacokinetics and pharmacodynamics of natural antioxidants, and assessing their therapeutic outcomes in glioblastoma patients.

## 7. Conclusions

Oxidative stress is increasingly recognized as a central driver of glioblastoma pathogenesis, progression, and resistance to therapy, with elevated ROS contributing to DNA damage, tumor proliferation, metabolic reprogramming, immune evasion, and therapeutic failure (Table 1). The hypoxic tumor microenvironment further amplifies oxidative stress through HIF-1α–mediated pathways, thereby fostering aggressive growth and deepening immunosuppression. Together, these mechanisms define redox imbalance as a core vulnerability in glioblastoma biology. Several signaling pathways, including SP/NK1R, and antioxidant regulators such as GPx1, SOD3, and TXNDC12, play critical roles in modulating redox dynamics and represent attractive therapeutic targets (Table 2 and Table 3).

Within this framework, antioxidants and nutraceuticals present promising yet complex opportunities for therapeutic intervention. These compounds can modulate redox-dependent signaling pathways, enhance apoptosis, sensitize glioblastoma cells to radiotherapy and chemotherapy, and in some instances help restore antitumor immune activity (Table 4). At the same time, their dual antioxidant–pro-oxidant potential underscores the necessity of careful patient stratification and precise dosing strategies to maximize benefit while minimizing unintended effects.

Recent advances in drug delivery technologies—including nanoparticles, liposomes, and redox-responsive carriers—have begun to address key limitations of bioavailability and blood–brain barrier penetration, thereby strengthening the translational potential of antioxidant-based approaches. Combination regimens that incorporate such compounds alongside standard therapies or novel nanomedicine platforms offer particularly compelling strategies to exploit the oxidative vulnerabilities of glioblastoma.

Taken together, current evidence positions oxidative stress not only as a pathogenic hallmark but also as a therapeutic opportunity. By bridging mechanistic insights into ROS-driven tumor biology with the clinical development of antioxidant interventions, future research can more effectively integrate redox modulation into comprehensive, personalized treatment strategies aimed at overcoming resistance and improving outcomes for patients with this devastating disease.

## Figures and Tables

**Table 1:** Oxidative stress in glioblastoma pathophysiology–key mechanisms.

Mechanism	Description	References
DNA Damage and Mutagenesis	ROS induce formation of 8-oxo-dG, leading to G→T transversion mutations	[23,33,46,47,48,49,50,51]
Tumor Cell Proliferation and Survival	ROS modulate signaling pathways (e.g., MAPK) that support tumor growth	[30,33,34,37,38,39,40,41,42,43,57,58,59,60,61,62,63,64]
Hypoxia-Driven ROS Production	Hypoxia increases ROS via HIF-1α signaling, promoting migration and invasion	[68,69]
Immune Evasion	Oxidative stress promotes M2-like macrophage infiltration and suppresses NK cell activity	[75]
Therapy Resistance	ROS contribute to selection of resistant glioma stem cells; antioxidant adaptation confers resistance	[65,92,93]
Redox Imbalance and Metabolic Reprogramming	Oxidative stress upregulates glycolytic enzymes and DNA repair proteins (e.g., PKM2, APE1)	[84]
ROS-Induced Cell Motility	Elevated ROS enhance glioma cell motility and mitochondrial activity	[91]

**Table 2:** Oxidative stress-related targets in glioblastoma.

Target	Function in GBM	Effect of Modulation	References
GPx1	Antioxidant enzyme detoxifying H_2_O_2_ under hypoxic conditions	Depletion increases apoptosis and reduces tumor growth	[69]
SOD3	Regulates macrophage phenotype in TME	Inhibition reduces M2-like macrophage polarization	[75]
TXNDC12	Maintains redox homeostasis and supports tumor cell proliferation	Knockdown induces oxidative stress and reduces tumor growth	[103]
NUDT1	Controls mitochondrial ROS and prevents lipid peroxidation	Knockdown impairs mitochondrial function and induces cell death	[107]
C/EBPβ	Transcriptionally activates NQO1 and GSTP1	Knockdown increases ROS, reducing proliferation	[123]
POLR2J	Regulates oxidative stress and unfolded protein response	Silencing enhances vorinostat cytotoxicity	[125]
ALDH1L2	Maintains NADPH levels and antioxidant capacity	Knockout increases ROS and oxidative imbalance	[126]

**Table 3:** Molecular and targeted pathways in glioblastoma involving oxidative stress.

Pathway or Target	Role in GBM	Therapeutic Implication	References
HIF-1α/SERPINE1 axis	Activated under hypoxia via ROS, enhances invasion and EMT	Targeting ROS or HIF-1α may disrupt hypoxia-driven malignancy	[68]
SP/NK1R pathway	Promotes ROS generation and inhibits antioxidant systems	Aprepitant (NK1R antagonist) reduces ROS and induces cell death	[80,81,82,83]
Thioredoxin and Glutaredoxin systems	Major redox regulatory enzymes affected by SP signaling	Restoration enhances antioxidant capacity	[81,82]
APE1/PKM2/ENPP2 crosstalk	ROS-induced expression promotes DNA repair and glycolysis	May contribute to invasion and therapy resistance	[84]
GGCT/Pyroglutamic acid	Linked to glutathione metabolism and protein aggregation in resistant cells	Marker of oxidative stress-related drug resistance	[92]
CMA (Chaperone-Mediated Autophagy)	Triggered by ROS; absent in resistant GBM cells	Enhancing oxidative stress restores sensitivity to TMZ	[93]
C/EBPβ–NQO1/GSTP1 axis	Regulates expression of antioxidant enzymes in GBM	Targeting transcriptional regulation of antioxidant genes	[123]
iNOS/NO pathway	Protects GBM cells from PDT-induced ROS damage	Inhibition enhances ROS-based photodynamic therapy	[127]

**Table 4:** Antioxidants–molecular targets (↑/↓—increased/decreased).

Antioxidant	Cellular Effects	Molecular Pathway Targets	Cellular Process Targets	Oxidative Stress Targets	References
Carotenoids–general	Cell cycle regulation, angiogenesis inhibition	Akt/PI3K/mTOR, PPAR, Wnt, NF-κB	cyclin/CDK, VEGF, MMPs	↑ ROS production	[149,150,151]
Astaxanthin	Reduced invasiveness	-	↓ MMP-2 expression, ↓ MMP-9 activity	-	[154]
Lycopene	Mitochondrial dysfunction	↑ ERK activation	↑ p53 expression	↑ ROS	[161,162]
Crocetin	Reduced invasiveness	↓ RhoA, inhibits AKT signaling	↓ MMP-9	-	[166]
Zeaxanthin	↑ Apoptosis, disrupted oncogenic signaling	-	↑ cleaved PARP, ↑ Caspase 3, ↓ VEGFR2 kinase	-	[169]
Catalase (CAT)	↓ Proliferation, ↑ resistance to TMZ/radiotherapy	-	↓ CAT in nucleus/mitochondria	↓ H_2_O_2_ levels	[129,145,146,147]
Coenzyme Q10	↓ Invasiveness, ↑ sensitivity to TMZ, potential cell death at high concentrations	-	ATP production via electron transport, preserves GFAP network	↓ ROS (low conc.), pro-oxidant (high conc.)	[170,171,172,174,175,176,177]
Curcumin	↓ Proliferation, ↑ apoptosis, ↓ invasion, ↓ GSC viability, G2/M arrest, ↑ autophagy	PI3K/Akt, MAPK, JAK/STAT, Shh, NF-κB, STAT3, Nrf2/HO-1	Rb, p53, BCL-2, LC3B-II, p62, ENO1, MMP2, PRKD2	↑ ROS	[178,179,180,181,182,183,184,185,187,188,189,190,191,192]
Quercetin	↓ Viability, ↑ apoptosis, ↓ migration/invasion, ↓ proliferation, ↓ tumor growth	GSK3β/β-catenin/ZEB1, p-STAT3, Rac1	Caspases, cleaved PARP, E-cadherin, N-cadherin, vimentin, Ki67, Axl, p66Shc	↑ ROS, ↑ SOD expression, ↓ ROS (via Rac1)	[199,200,201,202,203]
Fisetin	↑ Apoptosis, ↑ DNA damage, ↓ therapy-induced senescence	Nrf2–KEAP1 complex	↓ acHMGB1, ↑ TFEB, ↑ p53, ↓ DNA repair, ↑ γH2AX	-	[206,207]
Rutin	↓ Viability, ↓ proliferation, ↓ migration, microglial chemotaxis	STAT3	miRNA-125b, IL-6/IL-10, TNF, CCL2/5, CX3CL1, HBGF, IGF, GDNF	-	[208,209]
Hydrolized Rutin	↓ Proliferation, ↓ mitosis/necrosis, ↓ glioma grade	p53-independent mechanism	Cell cycle inhibition	-	[210]
Apigenin	↓ Viability, ↑ apoptosis, G2/M arrest, ↓ colony formation, ↓ migration	↓ p-AKT, ↓ p-mTOR, NF-κB p65	PARP cleavage, ↓ Bcl-xL, Bax, t-Bid, caspase-8/-9/-3, HIF-1α, GLUT-1/3, PKM2	↓ lactic acid	[212,213,214]
Acacetin	G2/M arrest, ↑ apoptosis (sub-G1)	-	↑ p21, ↓ Cyclin-A1/B1, ↓ Cdk-1	↑ ROS	[215]
Irigenin	G2/M arrest, ↓ proliferation, ↓ migration, ↓ tumor growth	↓ β-catenin signaling	↓ YAP	-	[216]
Kaempferol	↓ Viability, ↑ autophagy, ↑ pyroptosis, G2/M arrest, ↓ migration/invasion	↓ p-EGFR, ↓ p-SRC, ↓ p-STAT3	↑ gasdermin E, ↓ neurosphere formation	↑ ROS, ↓ mitochondrial potential	[217,218,219]
2-(3,4-dihydroxyphenyl)-5,7-dihydroxy-4H-chromen-4-one	↓ Cell viability	mTOR binding	-	-	[220]
5-hydroxy-3′,4′,6,7-tetramethoxyflavone	G0/G1 arrest, ↓ migration	-	-	-	[221]
Epigallocatechin gallate	↓ Proliferation, ↑ apoptosis, G1 arrest, ↓ migration/invasion	PI3K/AKT/mTOR	↓ GLUT1/4, ↓ PKM2, ↓ LDHV, ↑ Caspase-3, ↓ BCL-2, ↓ MMP-2/9, ↓ uPA/uPAR	-	[223,224,225]
Epicatechin	-	MDM2–p53 interaction inhibition	-	-	[226]
Chrysin	↓ Proliferation, ↓ migration/invasion, ↑ apoptosis	↓ p-ERK1/2 (MAPK), ↓ Nrf2	↓ HO-1, ↓ NQO-1	-	[227,228]
Xanthohumol	↑ apoptosis	PI3K/Akt–GSK3β–FBW7, ↑ p-ERK1/2, ↑ p-p38 MAPK	↓ HK2, ↓ c-Myc, ↓ Bcl-2, ↑ caspase-3/9, ↑ PARP cleavage	↑ ROS	[229,230]
Hispidulin	↑ apoptosis, inhibits TMZ-induced autophagy, ↑ TMZ sensitivity	-	↑ Bax, ↑ cleaved caspase-3/9, ↓ Bcl-2	-	[231]
Silibinin	↓ Proliferation, ↓ invasion, ↓ stemness, ↑ apoptosis, ↑ autophagy	PI3K, FoxM1, ↓ p-mTOR	↓ SLC1A5, ↓ YAP, caspase-3, PARP cleavage	-	[232,233]
Glutathione	↑ Cytotoxicity (when depleted), ↑ drug resistance, ↓ tumor growth	TERT–FOXO1–GCLC axis, cGAS–STING activation	↑ GS in mesenchymal GBM, ↑ GSR, ↑ GPX2	Neutralizes ROS, ↓ GSH (with auranofin), ↑ GSH synthesis	[135,136,137,138,139,140,141,142]
Resveratrol	↓ Viability, ↓ migration/invasion, ↑ apoptosis, G1 arrest, ↓ tumor growth	↓ NLRP3, ↓ JAK2/STAT3, ↓ AMPK–YAP, ↓ STAT3	↑ Bax, ↓ Bcl-2, ↑ Caspase-9, ↑ Pak2, ↓ MGMT, ↓ survivin, ↓ Ki-67, ↓ PD-L1	↑ ROS, ↓ SOD2, ↓ catalase	[238,239,240,241,242,243,244,245,246]
Rosmarinic Acid	↓ Tumor volume, ↓ invasion into normal brain tissue, ↑ apoptosis, ↓ proliferation, ↑ survival rates	-	-	↓ SOD activity, ↓ CAT activity, modulation of oxidative stress pathways	[248]
Sinularin	↑ Apoptosis, mitochondrial dysfunction, ↓ mitochondrial respiration capacity	-	↑ cleaved caspase 9, ↑ cleaved caspase 3, ↑ cleaved PARP	↑ ROS generation, ↓ SOD1/2, ↓ thioredoxin, compromised antioxidant defense	[250]
SOD3	Poor prognosis, ↓ immunosuppression (when knocked down)	M2-like macrophage transformation	-	↑ SOD3 expression	[75]
SOD2	Protects TMZ-resistant cells, ↓ ferroptosis	CYBB/Nrf2/SOD2 axis	-	↑ in mesenchymal GBM, protects from ROS/ferroptosis	[144]
Vitamin A	↓ Stemness, ↑ differentiation, ↑ apoptosis, G1/S arrest, ↓ invasion/migration	ERK1/2	↓ CD133, ↓ Sox2, ↑ GFAP, ↑ Bax:Bcl-2 ratio, ↑ cytochrome c, ↑ caspase-3, ↑ p27^kip1, ↓ CDK2, ↓ MMP-2/9	-	[253]
Vitamin E	↓ Proliferation, pre-G1 arrest, ↑ apoptosis, ↓ adhesion	PKC	↓ cyclin D1/E, ↑ Bax, ↑ TRAIL, ↑ caspase-3/8, ↑ cytochrome c, ↑ integrin, ↓ MDR1	↓ GSH, ↓ ROS, restores GSH levels	[253,259,260,261]
Vitamin C	↑ Ferroptosis, ↓ tumor growth, ↓ GSC self-renewal, ↑ TMZ/radiation sensitivity, ↑ autophagy	↑ p-Akt then ↓ Akt, ↑ JNK, ↓ HIF pathway, ↑ TET activity	↑ 5hmC, ↓ 5mC, ↑ H3K36me3, NSD1 promoter demethylation	↑ ROS, ↑ ferroptosis (with ferric iron)	[266,267,268,269,270]

## Data Availability

No new data were created in this study.
